# Genomic patterns in the widespread Eurasian lynx shaped by Late Quaternary climatic fluctuations and anthropogenic impacts

**DOI:** 10.1111/mec.15366

**Published:** 2020-02-23

**Authors:** Maria Lucena‐Perez, Elena Marmesat, Daniel Kleinman‐Ruiz, Begoña Martínez‐Cruz, Karolina Węcek, Alexander P. Saveljev, Ivan V. Seryodkin, Innokentiy Okhlopkov, Mikhail G. Dvornikov, Janis Ozolins, Naranbaatar Galsandorj, Milan Paunovic, Mirosław Ratkiewicz, Krzysztof Schmidt, José A. Godoy

**Affiliations:** ^1^ Department of Integrative Ecology Estación Biológica de Doñana (CSIC) Seville Spain; ^2^ School of Biological and Environmental Sciences Liverpool John Moores University Liverpool UK; ^3^ Mammal Research Institute Polish Academy of Sciences Białowieża Poland; ^4^ Department of Animal Ecology Russian Research Institute of Game Management and Fur Farming Kirov Russia; ^5^ Biological Faculty of Moscow State University Moscow Russia; ^6^ Laboratory of Ecology and Conservation of Animals Pacific Institute of Geography of Far East Branch of Russian Academy of Sciences Vladivostok Russia; ^7^ Far Eastern Federal University Vladivostok Russia; ^8^ Institute for Biological Problems of Cryolithozone Siberian Division of the Russian Academy of Sciences Yakutsk Russia; ^9^ Department of Hunting Resources Russian Research Institute of Game Management and Fur Farming Kirov Russia; ^10^ Department of Hunting and Wildlife Management Latvijas Valsts mežzinātnes institūts "Silava" Salaspils Latvia; ^11^ Institute of General and Experimental Biology Mongolian Academy of Science Ulaanbaatar Mongolia; ^12^ Natural History Museum Belgrade Serbia; ^13^ Institute of Biology University of Białystok Białystok Poland

**Keywords:** carnivore, Eurasian lynx, *Lynx lynx*, mitogenomes, phylogeography, population genomics

## Abstract

Disentangling the contribution of long‐term evolutionary processes and recent anthropogenic impacts to current genetic patterns of wildlife species is key to assessing genetic risks and designing conservation strategies. Here, we used 80 whole nuclear genomes and 96 mitogenomes from populations of the Eurasian lynx covering a range of conservation statuses, climatic zones and subspecies across Eurasia to infer the demographic history, reconstruct genetic patterns, and discuss the influence of long‐term isolation and/or more recent human‐driven changes. Our results show that Eurasian lynx populations shared a common history until 100,000 years ago, when Asian and European populations started to diverge and both entered a period of continuous and widespread decline, with western populations, except Kirov, maintaining lower effective sizes than eastern populations. Population declines and increased isolation in more recent times probably drove the genetic differentiation between geographically and ecologically close westernmost European populations. By contrast, and despite the wide range of habitats covered, populations are quite homogeneous genetically across the Asian range, showing a pattern of isolation by distance and providing little genetic support for the several proposed subspecies. Mitogenomic and nuclear divergences and population declines starting during the Late Pleistocene can be mostly attributed to climatic fluctuations and early human influence, but the widespread and sustained decline since the Holocene is more probably the consequence of anthropogenic impacts which intensified in recent centuries, especially in western Europe. Genetic erosion in isolated European populations and lack of evidence for long‐term isolation argue for the restoration of lost population connectivity.

## 1 INTRODUCTION

Climatic oscillations and geological events have influenced the range of species and the size and connectivity of their populations, driving divergence and admixture processes that give rise to the biodiversity patterns we see today (Avise et al., 1987; Endler, 1977). More recently, human‐driven habitat alteration, fragmentation and destruction, among other drivers of biodiversity loss, are fuelling the decline and subdivision of populations into small and isolated fragments where random genetic drift becomes the main evolutionary force. The result is often the loss of genetic variation, an increase in inbreeding in the population, and the genetic differentiation among populations (Benazzo et al., 2017; Srbek‐Araujo, Haag, Chiarello, Salzano, & Eizirik, 2018; Thatte, Joshi, Vaidyanathan, Landguth, & Ramakrishnan, 2018). Recent, human‐driven genetic divergence among populations must be considered together with the effects of long‐term evolution in isolation, which enable adaptive divergence and, eventually, speciation, as possible factors shaping current genetic patterns (Allendorf, Luikart, & Aitken, 2013; Frankham, Ballou, & Briscoe, 2002). It is thus important that the delimitation of conservation units and the design of conservation strategies are informed by good knowledge of the demographic and evolutionary processes that have acted upon the species across space and time.

Recently developed high‐throughput sequencing approaches can significantly expand our ability to obtain genomic‐scale information and infer evolutionary processes in nonmodel species in a cost‐effective way. Also, the availability of new reference genomes is helping to overcome most of the limitations of classical genetic markers and to expand the range of questions that can be addressed, including the assessment of the relative influence of current (human‐driven) and long‐term evolutionary processes (Abascal et al., 2016; Feng et al., 2019; Li et al., 2014; Murchison et al., 2012).

The Eurasian lynx (*Lynx lynx*) is one of the most broadly distributed felids in the world, representing a suitable but understudied model for exploring the long‐term, as well as recent anthropogenic impacts, patterns of variation in the genome. The species' range extends from Central Europe to the Asian Far East, encompasses a wide range of habitats (shrubland, forest, desert, rocky areas and grassland) and climates (Mediterranean, temperate, boreal; from sea level to 5,500 m), and includes populations with varied recent demography, some of which led to near extirpation in the last century by anthropogenic impacts and extermination policies, followed by varied rates of recovery. The fossil and historical records indicate that the Eurasian lynx was already present in Europe during the Pleistocene (Sommer & Benecke, 2006), and that its westernmost range reached the Iberian Peninsula (Clavero & Delibes, 2013; Rodríguez‐Varela et al., 2016) and Great Britain (Hetherington, Lord, & Jacobi, 2006). The species was extirpated from most of central, western and southern Europe during the 20th century, and the remaining central European populations are severely fragmented and isolated. Previous genetic studies of these European populations using microsatellite markers and mitochondrial DNA (mtDNA) sequences have found the lowest levels of diversity and strong population differentiation within Europe, but also relatively high levels of gene flow among populations across the central part of its range (Förster et al., 2018; Hellborg et al., 2002; Ratkiewicz et al., 2012; Schmidt, Kowalczyk, Ozolins, Männil, & Fickel, 2009). In contrast, the range of the species in Asia is often described as continuous, with demographically healthy and well‐connected populations (Rueness, Naidenko, Trosvik, & Stenseth, 2014), although the information is often scattered or completely lacking. The only genetic study that covered most of the distribution range of the species, by resorting to museum specimens, found three different mitochondrial clades and a clear structuring along an east–west gradient (Rueness et al., 2014). However, the low resolution imposed by the few microsatellites and the small mitochondrial region used hampered robust conclusions on the phylogeographical relationships among populations and on the ultimate drivers of the observed genetic differentiation. While the Eurasian lynx has historically been divided into many subspecies based mostly on morphological characteristics (Kitchener et al., 2018), so far genetic studies have not been able to provide data of sufficient resolution to resolve intraspecific taxonomy.

With the power of genomics and the recent availability of a reference genome from the closely related Iberian lynx (*Lynx pardinus*; Abascal et al., 2016), we analysed the genetic variation of the Eurasian lynx across most of its geographical range to assess the relative influence of evolutionary history and recent demographic declines and fragmentation. Specifically, we addressed to what extent long‐term isolation and/or recent human‐driven changes have impacted the lynx populations by analysing: (a) the history of population size, divergence and admixture among lynx populations; and (b) the current patterns of genetic structure and diversity across its distributional range. Additionally, we discuss the level of genetic support for the proposed subspecies and the implications for the conservation of its most endangered populations.

## 2 MATERIALS AND METHODS

### 2.1 Sampling

**Figure 1:**
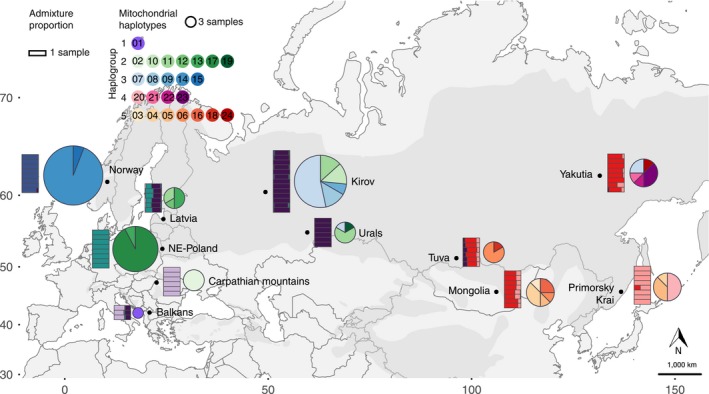
Distribution of mitogenomic and nuclear autosomal variation across Eurasian lynx populations. Pie charts represent the frequency of each of the 24 identified mitochondrial genome haplotypes in each population (right), and rectangles depict the ancestry of individuals in each of six genetic clusters, as estimated with NGSAdmix (left)

We sampled 80 *Lynx lynx* across the distribution range of the species, including five out of the six subspecies proposed by the IUCN Cat Specialist Group: *L. l. lynx*, *L. l. balcanicus*, *L. l. carpathicus*, *L. l. isabellinus* and *L. l. wrangeli* (Kitchener et al., 2018) (Figure 1; Table S1). Also, one *Lynx rufus* (bobcat) from Jerez Zoo (Spain), and one *Lynx canadensis* from Ostrava Zoo (Czech Republic) were sampled to be used as an outgroup and to identify the ancestral state of detected variants (Methods in Appendix S1).

The design of the study was intended to sample a minimum of six individuals from 11 a priori populations defined on the basis of only geography: (1) North‐Eastern (NE) Poland (Białowieża and Knyszyn Primeval Forests); (2) Balkans; (3) Carpathian Mountains; (4) Latvia; (5) Norway; (6) Kirov region, Russia; (7) Ural Mountains, Russia; (8) Tuva (the Republic of Tyva), Russia; (9) Yakutia (Republic of Sakha), Russia; (10) Primorsky Krai, Russia; and (11) Mongolia (for detailed descriptions of the populations and sampling methods see Methods in Appendix S1 and Table S2). These populations represent different climatic and land cover zones, but they also differ with respect to demographic history and recent human exposure. Based on available records of recent demographic status (see Methods in Appendix S1 and Table S2), the Norway, NE Poland, Carpathians and Balkans populations are the remnants of a process of anthropogenic range contraction in Europe initiated in the 16th century driven by habitat alteration and direct persecution, which intensified by the turn of the 19th and 20th centuries by the implementation of extermination policies in several countries. The remnant central European populations recovered from their rather extreme bottlenecks following legal protection enacted during the 20th century, but have remained relatively isolated until today. In more eastern and northern parts of Europe, Latvia, Kirov region of Russia and the Urals, populations remained moderately large and/or well interconnected during this process. In Asia, we sampled Tuva, Yakutia, Primorsky Krai and Mongolia, which are considered part of a large contiguous range that has been much less affected by habitat alteration (see Methods in Appendix S1 and Table S2).

### 2.2 DNA extraction, sequencing and mapping

Samples consisted of good quality tissue or blood, except for the Balkan samples, which were poorly preserved specimens that yielded signatures of low quality and extensive contamination (Methods in Appendix S1). All samples were digested overnight using proteinase K and genomic DNA (gDNA) was extracted using silica‐coated paramagnetic beads (NucleoMag Tissue, Macherey‐Nagel). Depending on the sequencing strategy (depth targeted and quality of the gDNA), library preparation and sequencing of the samples differed (details in Methods: Appendix S1). Briefly, gDNA was sheared, size‐selected, end‐repaired and adenylated following the appropriate Illumina protocol. After ligating indexed paired‐end adapters, DNA fragments were amplified via PCR (polymerase chain reaction) if required, and the quantity, quality and size of the libraries were assessed. Finally, libraries were sequenced using Illumina HiSeq2000 or Illumina HiSeq X‐10, in centro nacional de análisis genómico (CNAG) or Macrogen facilities, respectively. In all cases, samples were sequenced using Illumina protocols, and primary data analysis was carried out with the standard Illumina pipeline. We performed a quality control of our data, and we trimmed and mapped our sequences to a 2.4‐Gb *Lynx pardinus* nuclear reference genome, which diverges from the Eurasian lynx by an average of ~0.00122 substitutions per site (Abascal et al., 2016; http://denovo.cnag.cat/genomes/iberian_lynx/), using bwa‐mem (Li, 2013) (details in Methods: Appendix S1).

To reconstruct mitogenome sequences we used raw reads coming from both this whole‐genome (WG) project and a separate capture‐based study (16 additional individuals, E. Marmesat et al., unpubl. results). Reads were mapped to the *L. lynx* mitochondrial reference genome generated by Abascal et al. (2016) using bwa‐mem (Li, 2013) with default parameters. We called single nucleotide polymorphisms (SNPs) using freebayes (Garrison & Marth, 2012) and constructed a consensus for each mitochondrial genome using the FastaAlternateReferenceMaker command in gatk (McKenna et al., 2010) (Table S1; and further details in Methods: Appendix S1).

In summary, we generated WG resequencing data for 80 *L. lynx* individuals, 76 at low–medium depth (4–13×) and four at high depth (19–28×), and mitogenome data for 96 *L. lynx* individuals with an average depth of 137.7× (Table S1). For analyses including all individuals, data at medium–high depth were randomly subsampled to a depth within the range of the low–medium depth data using samtools view ‐s (Li et al., 2009) to avoid biases associated with differences in sequence depth. Samples from the Balkan population were used to assemble mitogenomes and determine global autosomal genetic structure (principal components analysis [PCA] and NGSAdmix), but we did not estimate diversity and neutrality indices from these samples, as we found an excess of changes probably associated with their suboptimal conservation (see Methods in Appendix S1).

Due to the focus of our study on demographic reconstruction and neutral evolution, we only considered intergenic regions, representing 61% of the nuclear genome (~1.5 Gb), for most of the analyses (further information on neutral regions definition in Methods: Appendix S1). We also identified nonrecombining parts of X and Y chromosomes that were excluded from analyses on the autosomes (i.e., PSMC [pairwise sequentially Markovian coalescent], measures of autosomal diversity) and used to compare patterns of diversity with the autosomes.

### 2.3 Data analysis

#### 2.3.1 Nuclear demographic and divergence reconstruction using PSMC

To infer changes in the effective population size (*N_e_*) through time (3 × 10^6^–10^4^ years) on the basis of the nuclear genome, we used a PSMC model (Li & Durbin, 2011). This model infers population size history from the distribution of the local density of heterozygous sites in a single diploid sequence. Therefore, for this analysis, we used autosomal whole genome data of four *L. lynx* individuals sequenced at higher depth (>19×), two from Asia (Vladivostok and Yakutia) and two from Europe (Carpathians and Kirov). For each individual, we generated a diploid consensus file using samtools mpileup as suggested by Li and Durbin (2011). For this analysis, minimum read depth was set to 7× and maximum read depth to 60× for all individuals.

To infer the divergence time between populations we built pseudodiploids by randomly combining haplotypes of two individuals sampled in different populations (Cahill, Soares, Green, & Shapiro, 2016; Chikhi et al., 2018). To do so, we used the fasta files generated from the bam files during the PSMC pipeline. First, we intersected the two fasta files to obtain the list of scaffolds represented in both samples, and we then used seqtk mergefa (https://github.com/lh3/seqtk) to randomly sample one allele from each of the two fasta Files. PSMC analyses were then conducted on the pseudodiploids as described above for true diploids. We performed 100 bootstraps (Li & Durbin, 2011) for both the original and the pseudodiploids analysis.

#### 2.3.2 Nuclear demographic reconstruction using stairway plot

We reconstructed recent demographic trajectories of the populations using stairway plot (Liu & Fu, 2015), which infers more recent histories than PSMC (5 × 10^5^–10^2^ years ago), although with limited power when sample sizes are below the hundreds (Beichman, Huerta‐Sanchez, & Lohmueller, 2018). The program is a model‐free method that uses the unfolded site frequency spectrum (SFS) to infer population size changes over time. We generated the SFS for each population using first angsd (Korneliussen, Albrechtsen, & Nielsen, 2014; Korneliussen, Moltke, Albrechtsen, & Nielsen, 2013; Li, 2011) to generate the sample allele frequency (SAF) for each population, and then realsfs (Korneliussen et al., 2013) to generate the population SFS.

#### 2.3.3 Nuclear demographic reconstruction using snep

Most recent changes in *N_e_* (<2 × 10^3^ years ago) were inferred using the relationship between linkage disequilibrium and *N_e_* (Hill, 1981) as implemented in the software snep v1.131 (Barbato, Orozco‐terWengel, Tapio, & Bruford, 2015). This method uses the genetic distance between markers for estimating *N_e_* in different periods. To increase the contiguity between markers we converted our SNP coordinates in the Iberian lynx assembly (41,700 scaffolds, *N*_50_ = 1.52 Mb) into cat coordinates (chromosomal level assembly) using the synteny previously defined by Abascal et al. (2016). After splitting our VCF into the different populations, we generated a map and ped file using plink 1.9 (http://www.cog-genomics.org/plink/1.9/; Chang et al., 2015). We used a recombination rate of 1.9 centimorgans (cM) Mb^−1^ (Li et al., 2016) and Sved and Feldman's (1973) mutation rate modifier for correcting the recombination rate. We also used the sample size correction for unphased genotypes.

PSMC, stairway plot and snep outputs were plotted, scaled to time and population sizes assuming a mean generation time of 5 years (Lucena‐Perez et al., 2018) and a mutation rate per site per generation of 6 × 10^−9^ (Abascal et al., 2016).

#### 2.3.4 Nuclear divergence and admixture reconstruction using treemix

We used treemix version 1.12 (Pickrell & Pritchard, 2012) to infer patterns of splits and admixtures between *L. lynx* populations. For this analysis, which requires a set of called variants, we followed the genome VCF (GVCF) workflow in gatk 3.4 (McKenna et al., 2010) (Methods in Appendix S1) on all our *L. lynx* populations (except the Balkans; excluded from treemix analysis), and on the one *L. rufus* sample as outgroup. Allele counts were extracted from each of the two VCF files using a custom script, and both resulting allele count files were merged under the assumption that any SNP absent in one of the species (but present in the other) would be fixed for the reference allele. We ran treemix version 1.12 (Pickrell & Pritchard, 2012) setting *L. rufus* as outgroup and the block size to 100. We modelled between zero and six migration events (0 ≤ *m* ≤ 6) and calculated the proportion of variance in relatedness between populations explained by each model. To assess the consistency of migration edges, we performed nine additional runs for *m* = 2 with different random seeds. Both the tree models and the residuals from the fit of the models to the data were visualized using the R script included in treemix.

We also ran the three‐population test (Reich, Thangaraj, Patterson, Price, & Singh, 2009), as implemented in the *threepop* program of treemix, to detect past admixture between populations. This test checks whether population X is related to populations A and B through a simple tree (in which case the *f* statistic, defined as the product of the frequency differences between A and X, and B and X, is expected to be positive), or through an admixture of A and B (where negative *f* statistic values are expected). To assess the statistical significance of the test, *threepop* obtains a standard error from blocks (here set to a size of 100 SNPs) and then generates a Z score. Z score values below −2 indicate significant support for admixture. This test was conducted for all possible combinations of (a) three representative European populations (Carpathians, Kirov and the Urals), (b) one Asian test population (Tuva or Yakutia) and (c) the three remaining Asian populations (Vladivostok, Mongolia, and Yakutia or Tuva depending on which was the test population).

#### 2.3.5 Nuclear genomic structure

To assess the genetic relationships among samples we performed a PCA. We calculated the genotype posterior probabilities using angsd (Kim et al., 2011; Li, 2011) and NGSTools/ngsPopGen/ngsCovar (Fumagalli, 2013; Fumagalli et al., 2013). For all the analyses using angsd the filters applied were: ‐uniqueOnly 1 ‐remove_bads 1 ‐only_proper_pairs 1 ‐baq 1 ‐C 50 ‐minMapQ 30 ‐minQ 20 ‐doCounts 1 –minInd (number of individuals in the population/2) –setMaxDepth (average [AVR] depth for the population + [0.95 * stdev depth for the population]) –setMinDepth (AVR depth for the population − [0.95 * stdev depth for the population]) − skipTriallelic 1); and we took the base observed in *L. rufus* as the ancestral state (more details on how we reconstructed our ancestral state in Methods: Appendix S1). For PCA, and also pairwise genetic distances and admixture analyses we set a SNP_pval of 1e−3. The resulting PCA was plotted using scatterplot3js from threejs library in R (Lewis, 2017). PCA coordinates were scaled to geographical coordinates (Procrustes analysis, following Borg & Groenen, 1997) to assess similarities between geographical and genetic distribution using the package mcmcpack (Martin, Quinn, & Park, 2011) in R (R Core Team 2019).

We used the genotype likelihoods calculated with angsd (Kim et al., 2011; Li, 2011) to perform a structure analysis using NGSadmix (Li, 2011; Skotte, Korneliussen, & Albrechtsen, 2013). We ran NGSadmix with a range of a priori populations from *K* = 1 to *K* = 13. The analysis was rerun 10 times to evaluate convergence and results were plotted using R. We used clumpak (Kopelman, Mayzel, Jakobsson, Rosenberg, & Mayrose, 2015) to evaluate the optimal *K* following Evanno, Regnaut, and Goudet (2005). We used the identified genetic clusters to confirm that all our a priori defined populations were genetically homogeneous (i.e., all the individuals showed similar ancestry proportions and therefore could be pooled for population‐based analysis).

#### 2.3.6 Nuclear genomic differentiation among populations

A two‐dimensional unfolded site frequency spectrum (2d‐SFS) was computed using realsfs (Korneliussen et al., 2013) for each population pair. 2d‐SFS and SAF files were used as priors to calculate *F*_ST_ using realsfs (Korneliussen et al., 2013). To graphically visualize the genetic relationship among populations, we constructed a neighbour‐joining tree based on the pairwise *F*_ST_ matrix using the ape package in R (Paradis, Claude, & Strimmer, 2004).

To evaluate the influence of distance on genetic differentiation patterns we calculated the genetic distance among pairs of individuals using angsd and ngsdist from ngstools (Fumagalli et al., 2013; Korneliussen et al., 2014). Geographical distances among sampling points were calculated from their geographic coordinates using the Point Distance tool and Winkel Tripel projection in arcgis 10.5 (Esri, 2011). All distances involving samples from Norway were measured via the Scandinavian isthmus to estimate overland distance. Based on our previous results on PCA and admixture proportions, we plotted geographical distance against genetic distance splitting our results depending on whether the comparison was between two samples from Asia, two samples from Europe, or one sample from Europe and one from Asia. We tested the significance of these correlations with Mantel tests using the package vegan (Oksanen et al., 2018) in R. We also performed a partial Mantel to test the effect of a possible geographical barrier between Asian and European populations by introducing a binary variable coded as 0 when the two samples were from the same region and as 1 otherwise, and tested the effect of this variable while accounting for the effect of geographical distance.

#### 2.3.7 Nuclear genomic diversity

We calculated the genetic diversity (nucleotide diversity [π] and Watterson estimator [θ]) and Tajima's *D* neutrality index for each population. For autosomal and X chromosomes, we used angsd (Korneliussen et al., 2014; Korneliussen et al., 2013; Li, 2011) and realsfs (Korneliussen et al., 2013) to calculate diversity indices per site for each population (Korneliussen et al., 2013). Using thetastat (Fumagalli, Vieira, Linderoth, & Nielsen, 2014), we performed a sliding‐window approach with a window size set to 50,000 bp and a step size of 50,000. We classified our windows as autosomal or X chromosome if all the sites of the given windows belonged to either of these categories (Methods in Appendix S1). For comparison among populations, only windows with information for all the populations were used (828 X chromosome, and 24,392 autosomal windows). Sample size of the X chromosome differs from that of autosomal chromosomes (which is 2 × number of individuals), as it depends on the number of males and females sampled in the population (2 × number of females + number of males). Therefore, we recalculated our Watterson estimator (θ) by adjusting the correction factor (that accounts for sample size) to the actual sample size of the X chromosome in each population. Standard errors were calculated by bootstrapping over windows as implemented in the *boot* package for R (Canty & Ripley, 2017; Davison & Hinkley, 1997), to account for the correlation among nearby sites due to linkage disequilibrium (LD).

To infer recent population size changes we compared X chromosome θ versus. autosomal θ, while controlling for divergence (Pool & Nielsen, 2007). Divergence (*D*) was computed as the number of substitutions between *L. lynx* and *L. rufus* divided by the number of covered sites, based on a genus‐wide variant‐calling performed following the GVCF workflow in gatk 3.4 (McKenna et al., 2010) (Methods in Appendix S1), using one sample of each of the four species that comprise the genus *Lynx* (*L. lynx*, *L. pardinus*, *L. canadensis* and *L. rufus*). The ratio between diversity and divergence (θ/*D*) was used as a measure of diversity normalized by mutation rate. The average θ/*D* ratio was calculated for X chromosome (X) and autosomal (A) windows and these were used to obtain an X/A ratio for each population. Standard errors of X/A ratios were calculated by bootstrapping over windows. We used R to plot SFS and diversity values, as well as to plot our diversity estimates along with those reported for other mammals for comparison.

For the Y chromosome, we assigned 71 contigs, adding up to 33,032 bases, by applying our strict sequence depth criteria; that is, 90% of the total bases in a contig have a female/male ratio depth below 0.3, and an average normalized depth for males between 0.2 and 0.8 (Methods in Appendix S1, section Chromosome X and Y regions definition and molecular sexing). We performed SNP calling using freebayes, as previously done for the mitogenome, but no SNPs were called under standard quality filters, suggesting an overall lack of variation in this chromosome, as previously reported by Hellborg and Ellegren (2004) on the basis of 2040 bp of noncoding genic sequences.

#### 2.3.8 Mitogenomic analyses

Consensus mitogenome sequences were aligned and collapsed into distinct haplotypes using the pegas R package (Paradis, 2010). The number of segregating sites (*S*), haplotype diversity (*Hd*), nucleotide diversity (*π*) and mean number of pairwise nucleotide differences (*k*) were calculated using the popgenome (Pfeifer, Wittelsbuerger, Ramos‐Onsins, & Lercher, 2014) and ape (Paradis et al., 2004) R packages. Pie chart nodes representing the respective haplotype frequency in each of the populations (Figure 1) were calculated using R (R Core Team 2019). Phylogenetic relationships among haplotypes were inferred by constructing a median‐joining haplotype network (Bandelt, Forster, & Röhl, 1999) and represented in popart (Leigh & Bryant, 2015) coloured by population. R was used to plot *k* values for different mammal species for comparison.

To estimate a substitution rate for the mitochondrial genome in felids, we used a set of 15 felid mitogenomes downloaded from GenBank and beast version 2.4.8 (Bouckaert et al., 2014 see Methods in Appendix S1 for details). We incorporated the clock rate estimated as the substitution rate in the beast analysis of the 24 *L. lynx* mitogenome haplotypes obtained in our study (Bouckaert et al., 2014). For the intraspecific analyses, we used an ensemble of tRNAs, rRNAs and 12 genes using the HKY + G site model with a single partition. We used a strict clock and a coalescent constant population tree model, adequate when dealing with intraspecific sequences. The Markov chain Monte Carlo (MCMC) was run for 20 million steps. treeannotator version 2.4.8 was used to obtain the Maximum Clade Credibility tree after discarding 10% of initial trees as burn‐in. Results were visualized with figtree version 1.4.3 (http://tree.bio.ed.ac.uk/software/figtree/).

## 3 RESULTS

### 3.1 Demographic and divergence history based on autosomal data

**Figure 2:**
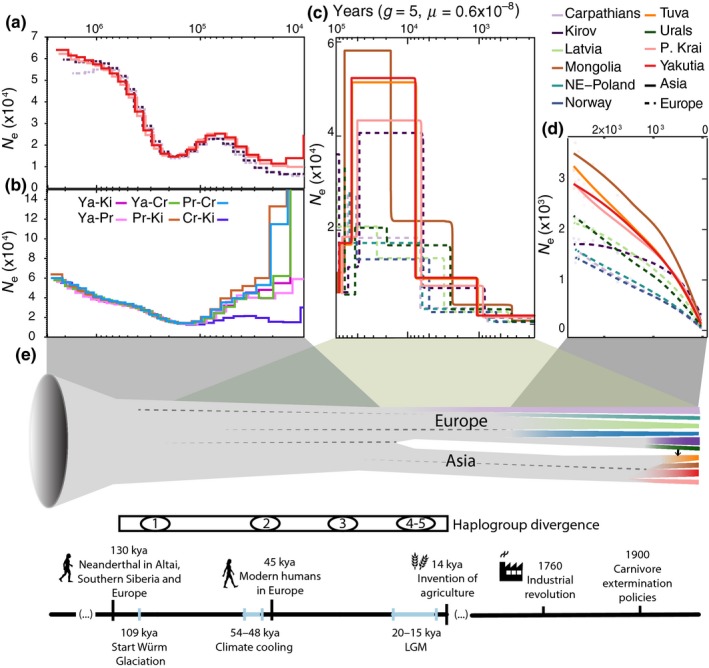
(a) Demographic reconstructions inferred with PSMC on the basis of autosomal data from one individual from Kirov, Yakutia, Primorsky Krai and Carpathians. (b) Pseudodiploid trajectories on PSMC. Sequences created by combining haplotypes from two different populations. Sudden population increases inferred for pseudodiploids are interpreted as the time of complete isolation of the two populations. (c) Demographic reconstruction inferred by stairway plot for all populations. (d) Recent demographic reconstruction inferred by snep for all populations. (e) Timeline of Eurasian lynx main demographic events, including partial (dashed lines) and complete isolation (branching) and admixture (arrow) between populations, along with haplogroup divergences, major climatic fluctuations and human milestones

Demographic histories inferred from the WG sequences of four *Lynx lynx* individuals (from the Carpathian Mountains, Kirov region, Yakutia and Primorsky Krai populations) using PSMC are concordant throughout most of the reconstructed period, indicating a long span of shared history between all populations (Figure 2a; Figure S1). *Lynx lynx *shows a long period of soft decline from 3 million years ago (Mya) to 600 thousand years ago (kya), and then declining more steeply to around 200 kya. This steep decline is followed by an apparent recovery of the population until 70 kya, although this could instead indicate the emergence of population structure (Chikhi et al., 2018; Mazet, Rodríguez, Grusea, Boitard, & Chikhi, 2016). From that point on, the demographic trajectories of European and Asian lynxes start diverging, with the European populations experiencing a sharper decline in *N_e_* than the Asian ones (Figure 2a; bootstraps presented in Figure S1). Accordingly, pseudodiploids constructed by combining Asian–European haplotypes start rising over the trajectory of true diploids around 100 kya, indicating the emergence of population structure, until they sharply increase around 15–20 kya (Figure 2b; Figure S1), indicating the time of complete population isolation between European and Asian populations. Unlike Asian–European pseudodiploids, we did not observe a sudden and sharp increase indicative of complete isolation in pseudodiploids consisting of two Asian or two European haplotypes. However, the moderate increase in population sizes with respect to real diploids suggests the emergence of some structure in Asia following the split of Asian and European populations, and little or no structure in Europe (at least) before 10 kya.

More recent demographic trajectories inferred for all the populations using stairway plot and snep are broadly congruent with the pattern inferred by PSMC (Figure 2c). stairway plot reconstructs a steep decline process during the last few millennia, with populations from Europe showing consistently smaller population sizes than Asian populations while snep reconstructs a smoother population decline spanning the last two millennia again with European populations showing smaller *N_e_* (Figure 2d). Both reconstructions show a more moderate decline for the European Kirov region population.

**Figure 3:**
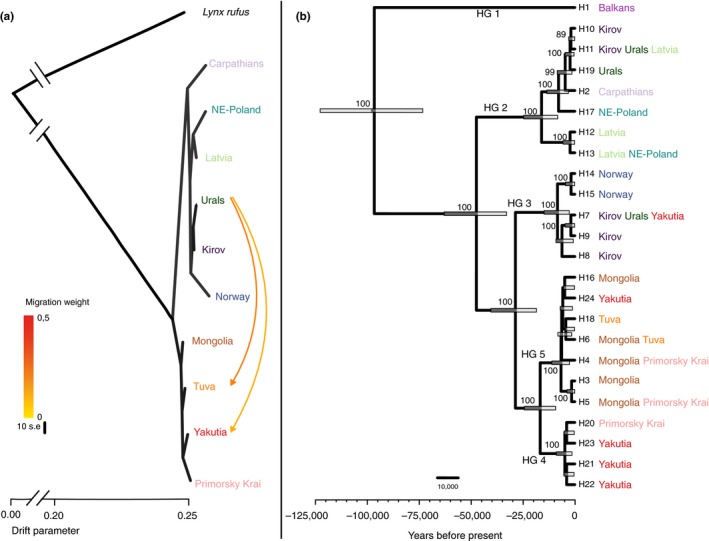
(a) Population tree inferred with treemix from nuclear autosomal data. Each arrow represents a migration event with its weight specified through the colour scale. (b) Bayesian maximum clade credibility tree inferred with beast depicting the relationship among mitogenome haplotypes. Numbers on nodes represent their posterior probability; only those over 0.85 are depicted

Considering all populations, the treemix analysis based on autosomal intergenic SNPs and using the bobcat (*Lynx rufus*) as outgroup supported a model of population divergence with one or two migration events (Figure 3a). In line with the PSMC results for pseudodiploids, the most basal split separates a European and an Asian population group, each with some shallower internal structure (Figure 3a; Figure S2). In the European group, Norway, NE Poland, and Carpathians show an increased drift parameter, indicating their larger differentiation compared to the rest of the populations.

Regarding gene flow, the treemix results support significant post‐divergence gene flow from the Urals to Tuva population (*m* = 1), or from the Urals to Tuva and to Yakutia population (*m *= 2; Figure 3a; residuals presented in Figure S3). Both these migration edges consistently appear in all nine independent runs at *m* = 2 (Figure S4), and they are still supported in tree models with three to six migration events (Figure S2; see fraction of variance explained by each of the models in Figure S5). Significantly negative *Z* scores (*Z* < −2) resulting from *f* statistics using *threepop* confirmed the admixture in Tuva, but not in Yakutia (Table S3), and identified all western and eastern populations, except Yakutia, as putative sources, with more negative *Z* scores the closer is the western source population (Ural Mountains < Kirov < Carpathian Mountains).

### 3.2 Mitogenomic divergence

Unlike the pattern inferred for the nuclear genome, the phylogenetic tree reconstructed from whole mitogenome sequences revealed several old mitochondrial lineages in Europe, which may have diverged during periods of isolation in separate glacial refugia (Figure 3b). The oldest split was dated around 96.5 kya (95% confidence interval [CI]: 73–122 kya), and separates the most divergent haplogroup, haplogroup 1 (46 out of 89 segregating sites), currently restricted to the Balkan population. A second mitogenomic split, dated around 47.4 kya (95% CI: 32–62 kya) defines haplogroup 2, whose current distribution includes the Carpathian Mountains and Baltic states populations. A subsequent split around 28.6 kya (95% CI: 18–40 kya) separated haplogroup 3, occurring mostly in northern and eastern Europe, from all the Asian haplogroups (4 and 5). Finally, the split between haplogroups 4 and 5 around 17 kya (95% CI: 9–24 kya) is coincident with an internal diversification within haplogroup 2.

### 3.3 Current nuclear genetic structure

Our a priori populations vary in the extent of the area sampled, ans may have affected our estimations of genomic diversity and differentiation. Nevertheless, individual‐based clustering and PCAs confirmed the relative genetic homogeneity within and differentiation among our a priori defined populations (i.e., ancestry composition is similar among individuals of the same population and different between populations).

**Figure 4:**
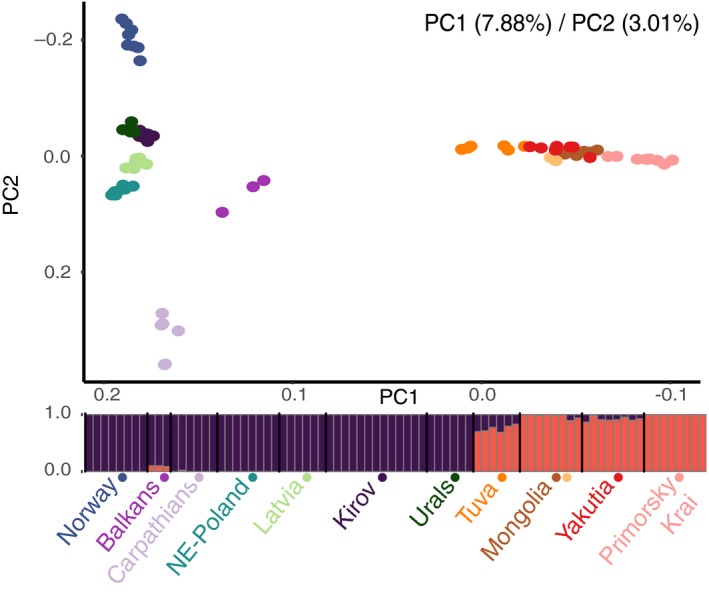
Relationship among individuals based on nuclear autosomal genotypes. PCA separates eastern and western individuals in the first axis (7.88% of the variance explained), and westernmost populations in the second axis (3.01% of the variance explained). Individual ancestry in each of the two clusters defined in the NGSAdmix analysis. Populations are sorted from west to east. Two different colours represent the Mongolia population, as this population comprises two different habitats (orange representing Ömnögovi, and brown Central and Khentii Aymag)

In agreement with the historical divergence and demographic processes shown in the previous section, nuclear population data reveal a contemporary major division between individuals in Asia and Europe. Both PCA and NGSAdmix separate these clusters first: PC1, *K* = 2, supported as the uppermost level of structure by the Evanno et al. (2005) method (Table S4) (Figure 4, Figure S6; interactive version available as supplementary material). Two populations show some admixed ancestry in these analyses: Balkan individuals with some Asiatic ancestry and individuals from Tuva (as well as, to a lesser extent, individuals from Yakutia) with some European ancestry, the latter supporting the historical admixture inferred by treemix. The historical isolation of European and Asian populations is also supported by a larger genetic distance between pairs of Asia–Europe individuals than that expected due solely to geographical distance (Figure S7).

The second and third axis in the PCA, and subsequent partitions in the structure analysis (*K* = 3, *K* = 4 and *K* = 5), separate bottlenecked populations in Europe (Carpathian Mountains, Norway and NE Poland) (Figure 4, Figures S9–S11; File S1). The high differentiation exhibited among European populations (average pairwise *F*_ST_ = 0.210; Figure S8, Table S5) contrasts with the relative homogeneity in Asia (average pairwise *F*_ST_ = 0.098; Figure S8, Table S5), where only the easternmost population of Primorsky Krai stands out as a separate cluster in some runs at 3 ≤ *K* ≤ 6 (Figure 1; Figures S9–S12). Accordingly, when PCA coordinates are projected onto a map, European populations show a major distortion in the projection compared to Asian ones, suggesting an isolation by distance scenario in the case of Asian populations, but a higher differentiation that is not explained solely on the basis of distance for the westernmost populations (Figures S13 and S14). The greater differentiation associated with bottlenecked populations (NE Poland, Norway and Carpathians) is also observed in isolation by distance plots, where the inclusion of bottlenecked populations increases the slope of the regression line (Figure S15).

### 3.4 Autosomal genetic diversity

**Figure 5:**
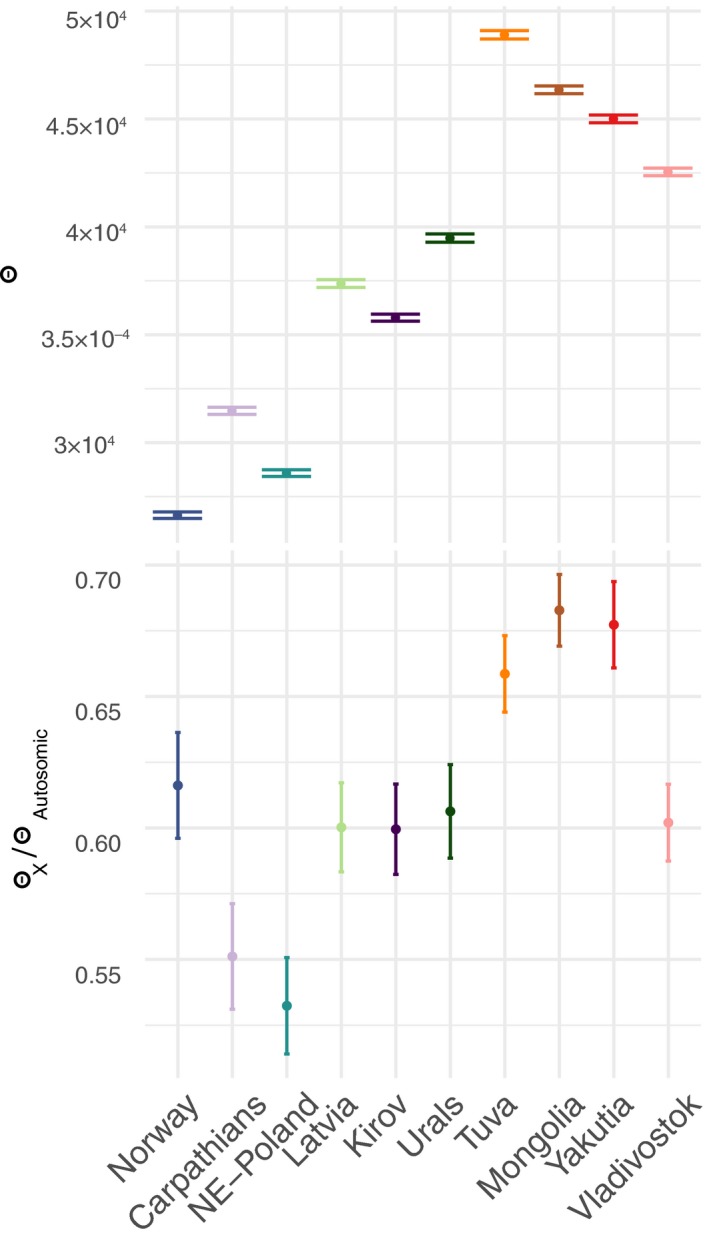
Waterson's theta (Θ) values for autosomal sites and ratio of Θ diversity in X chromsome versus. autosomes for the different populations. Populations are sorted from west to east

Genetic diversity levels in Eurasian lynx are in the low range of those reported based on genome‐wide data for other mammals, including some rare and endangered populations (Figure S16). This is especially the case for the bottlenecked European populations: NE Poland, Carpathian Mountains and Norway (Figure 5; Figures S16 and S17, Table S6). These westernmost populations also show a flatter site frequency spectrum compared to the rest of the populations (Figure S18), and a significantly positive Tajima's *D* value (Table S6), both indicative of a recent reduction in population size. NE Poland, followed by the Carpathians, also show the lowest X/A ratio, an additional indication of a recent, severe population size reduction (Pool & Nielsen, 2007). The X/A ratio in Norway is, however, larger and comparable to that found in the Ural Mountains, a pattern probably related to its subsequent growth (Pool & Nielsen, 2007). Conversely, the Urals and the Kirov regions show the highest diversity within Europe (Θ and π, respectively), but are exceeded by all Asian populations, with the highest genetic diversity present in Tuva despite the relatively small geographical area sampled (Figure 5; Figure S17).

### 3.5 Mitochondrial structure and diversity

In agreement with the autosomal data, overall mitogenomic diversity at the species level is very low (Figure S19). Only 89 segregating sites, out of 16,449 sites sequenced, defined the 24 haplotypes found (0.54%; Tables S7 and S8). We observed several populations in which European haplogroups 2 and 3 (e.g., Kirov, the Urals) or Asian haplogroups 4 and 5 co‐occur (e.g., Yakutia) (Figures 1 and 3; Figure S20), but we detected only one instance of the occurrence of a typically European haplotype (haplotype 7, haplogroup 3) in an Asian population (Yakutia), and none in the opposite direction. The westernmost bottlenecked populations also harbour lower diversities in the mitochondrial genome: the Carpathians and the Balkans show no mitogenomic diversity at all, while Norway and NE Poland populations also have low haplotype diversity (*H*_d_ = 0.12 and 0.15, respectively; Figure 1; Figure S20, Table S7); populations in Asia (Yakutia and Mongolia) show the highest haplotype diversity (*H*_d_ = 0.86 in both cases; Figure 1; Figure S20, Table S7).

## 4 DISCUSSION

Here we report the results of the most comprehensive analyses to date of the Eurasian lynx's evolutionary history and contemporary genetic variation, considering the extent of sampling both across the species range and across the genome. Our results indicate that the Eurasian lynx was genetically quite homogeneous at least until 100 kya, when lynx populations started diverging and the species entered a widespread and continuous demographic decline that affected the European populations in particular. Lower population sizes and increased fragmentation in the westernmost part of the distribution in more recent times probably drove the genetic differentiation between European populations that are otherwise geographically and ecologically close. Conversely, and despite the large range and the wide diversity of habitats, we observed a highly homogeneous genetic pattern among Asian populations, compatible with an isolation by distance pattern. Climatic oscillations during the Late Pleistocene, together with an increasing human pressure especially after the Last Glacial Maximum (LGM) which ended up with the extirpation of many European populations, probably shaped the current genetic patterns of the species.

### 4.1 Pleistocene

Phylogenetic and demographic analyses support a long common history for Eurasian lynx populations during most of the Pleistocene. The species suffered a massive demographic decline registered by PSMC between 3 Mya and 200 kya (Figure 2a), which may reflect a founder effect associated with the speciation from a common ancestor of Eurasian and Canada lynxes (recently dated around 1–1.2 Mya; Li, Figueiró, Eizirik, & Murphy, 2019). Low species‐wide genetic diversity, which is comparable to that of the white African lion or Greenlandic brown bear (Figure S16), might be—at least partially—the long‐term consequence of this drastic reduction in population size (Frankham, 2015; Frankham et al., 2011). From 200 to 70 kya, PSMC shows an apparent increase in population size, after which the species entered a continuous population decline (Figure 2a). This transient population size increase probably instead indicates the emergence of population structure (Chikhi et al., 2018; Mazet et al., 2016), as suggested by the departure of demographic trajectories of pseudodiploids in PSMC, and by the deepest divergence of mitochondrial haplogroups around 100 kya (Figures 2b and 3b). The isolation between eastern and western populations became complete around 22–15 kya, coinciding with the LGM (20–15 kya), and with the time of the most recent split that is registered in both nuclear and mitogenomic data (Figures 2 and 3). At a nuclear level, clustering and differentiation analyses reflect a basal divergence between Asian and European populations (Figures 1 and 4), and indicate that the genetic differentiation between individuals from the two clusters is larger than expected based solely on geographical distance (Figure S7). This east–west axis of differentiation was previously reported by Rueness et al. (2014). The lack of clear geographical barriers for lynxes during this period supports the idea that the emergence of the structure was preceded by a range contraction. For instance, barriers previously identified for small mammals between the two continents, including the Yenisei river (Kohli, Fedorov, Waltari, & Cook, 2015) and the Ural Mountains (Brunhoff, Galbreath, Fedorov, Cook, & Jaarola, 2003), are unlikely to act as strong barriers for such a large and mobile carnivore (Zimmermann & Breitenmoser, 2007).

Within continents, European populations underwent periods of isolation during the Late Pleistocene, as revealed by the divergence of different mitochondrial lineages, whereas populations in Asia remained largely connected as shown by nuclear and mitochondrial data (Figures 2b and 3b).

#### 4.1.1 Climatic impacts during the Pleistocene

Climatic fluctuations during the Late Pleistocene could have contributed to range contraction and subsequent emergence of population structure and divergence as revealed by nuclear and mitochondrial data (Figures 2 and 3). Accordingly, the date of the complete isolation between the Asian and European nuclear genetic groups is coincident with the LGM (20–15 kya) (Schlolaut et al., 2017; Svendsen et al., 2004) (Figure 2e). Older periods of isolation and divergence due to climatic oscillations during the Pleistocene are only registered in mitogenomic patterns, with the divergence of different haplogroups coinciding with the start of glaciation periods (HG1 divergence, 96.5 kya, start of Würm glaciation period [Ice Age], 109 kya), or significant global climate cooling (HG2 divergence, 47.4 kya, 48–54 kya [Kindler et al., 2014]; HG4–HG5 divergence and internal diversification of HG2, 17 kya, LGM) (Figure 2e). At least two of these haplogroups, HG1 and HG2, seem to have diverged in separate glacial refugia, the Balkans and the Carpathians, respectively, as previously suggested for lynx and other temperate mammals (Anijalg et al., 2018; Bilton et al., 1998; Gugolz, Bernasconi, Breitenmoser‐Würsten, & Wandeler, 2008; Ratkiewicz et al., 2014; Schmitt & Varga, 2012; Sommer & Nadachowski, 2006; Taberlet, Fumagalli, Wust‐Saucy, & Cosson, 1998). HG3, now present mainly in northern and eastern Europe, suggests a possible additional northeastern refugium for the species in Europe.

Deep mitochondrial divergences contrast with the recent isolation within Europe inferred from nuclear data. This discrepancy could be attributed to male‐biased dispersal, which is characteristic of the species (Holmala et al., 2018; Schmidt, 1998). Faster and deeper divergence of mitogenomes during periods of isolation followed by admixture between European populations, driven mostly by males, during the interglacial periods could have led to the pattern that we observe today. For instance, nuclear data suggest the existence of gene flow between the Carpathians and Kirov at least until 10 kya (Figure 2), while the divergence of the mitogenomic haplogroups typical of these populations was dated to ~50 kya (Figure 3). The co‐occurrence of different haplogroups in different populations suggests that, to a minor extent, females also contributed to the admixture of the populations. For instance, the Carpathians population with a unique haplotype (2), which is basal to HG2, could have acted as a source for postglacial colonization of this haplogroup northward and eastward, as suggested by the fact that HG2 is also present in populations such as Kirov, Urals or Latvia. Similar scenarios of isolation in refugia during glacial periods followed by colonization during interglacials have been described for species with similar habitat requirements, such as brown bear (Anijalg et al., 2018) or grey wolf (Pilot et al., 2010; Vila et al., 1999).

#### 4.1.2 Anthropogenic impacts during the Pleistocene

Besides climatic fluctuations, demographic declines during the Late Pleistocene could also be influenced by hominid species that were probably already widespread across Eurasia around 80–60 kya (Oppenheimer, 2012; Timmermann & Friedrich, 2016), and more dramatically by modern humans, who arrived in Eurasia around 45 kya and replaced other human populations, exceeding their population size by one order of magnitude (Mellars & French, 2011; Timmermann & Friedrich, 2016; Yang et al., 2017) (Figure 2e). Direct human impacts on lynx species have been documented during the Late Pleistocene, where a bone remain found in an Upper Palaeolithic site in the Iberian peninsula revealed the use of lynx as meat (Yravedra, 2005). Similarly, bones of leopard (*Panthera pardus*) have been found associated with hunting by prehistoric humans throughout Europe (reviewed by Sommer & Benecke, 2006). Additionally, the decline of ruminants since 100–50 kya has been partially attributed to human activities, rather than climatic oscillations (Chen et al., 2019), and hence the lynx decline during the Late Pleistocene could also be indirectly attributable to humans through negative effects on prey. Our hypothesis is in line with previous work that supports the idea that anthropogenic impacts in combination with climatic oscillations were one of the main drivers of the decline, and in some cases the extinction, of fauna and flora during the Late Pleistocene and early Holocene (Braje & Erlandson, 2013; Chen et al., 2019; Gretzinger et al., 2019; Lorenzen et al., 2011).

### 4.2 Holocene

The population decline of the species continued after the LGM with some differences among populations (Figure 2). The sustained negative population trends during the Holocene probably contribute to the signals of recent bottlenecks, such as reduced X/A ratios and high Tajima's *D* values that all populations show. Additionally, even the most diverse populations show values of genetic diversity similar to that of the severely bottlenecked Apennine brown bear (Benazzo et al., 2017), and only twice that of the extremely eroded sister‐species—the Iberian lynx (θ = 2.22 × 10^−4^, π = 2.6 × 10^−4^; Abascal et al., 2016), whose values are comparable to the least diverse Eurasian lynx populations (Figure S16). Still, differences in recent demography between populations, with European populations experiencing a severe reduction in population size throughout the Holocene and Asian populations usually maintaining a softer population decline, are reflected in current genetic patterns: European populations, especially westernmost ones, show larger genetic differentiation, increased drift parameters in treemix analysis, along with bottleneck signals and lower genome diversity. (Figures 4 and 5; Figures S8–S12, S17; Tables S5 and S6). Patterns of low diversity and high differentiation were previously reported for NE Poland (Ratkiewicz et al., 2012, 2014; Schmidt et al., 2009), as well as for Scandinavia and the Carpathians, using nuclear microsatellite markers and short mitochondrial sequences (Ratkiewicz et al., 2012, 2014) or an SNP set enriched for coding sequences (Förster et al., 2018). In contrast to westernmost populations, the current structure among Asian populations is shallow, similar to that found for Canadian lynx (*F*_ST_ = 0.09–0.10; Meröndun, Murray, & Shafer, 2019), the overall pattern is compatible with an isolation by distance scenario (Figure S14) and there is little support for more than one genetic cluster. Quite homogeneous genetic patterns across Asia are striking given the range of habitats occupied (e.g., from semidesert in Omnogovi, Mongolia, to boreal forest–tundra in Yakutia), and the several previously defined subspecies in this region.

#### 4.2.1 Anthropogenic impacts during the Holocene

The invention of agriculture produced a rapid human demographic expansion that started after the LGM in Europe, but only a few millennia ago in Asia (Gignoux, Henn, & Mountain, 2011; Nielsen et al., 2017; Skoglund et al., 2014), which eventually resulted in the emergence of urbanized and industrialized nation‐states. A more extensive, contiguous and less anthropogenically altered habitat in Asia during the Holocene might have contributed to the homogeneous genetic pattern in the continent, while in Europe, higher anthropogenic pressure, intensified in recent times as documented in historical records (Table S2), resulted in genetically structured and eroded populations. Carnivore extermination policies at the turn of 19th and 20th centuries extirpated the species from most of central Europe. Subsequent protection prevented total extirpation in remnant populations and allowed some recovery, but today lynx populations in this region remain highly isolated from each other and from the more contiguous range further north and east (Hellborg et al., 2002; Ratkiewicz et al., 2012; Schmidt et al., 2009). Demographic declines and genetic isolation during the last century have been particularly intense in Norway (together with neighbouring Sweden) and NE Poland. In Norway, the population was restricted to a few survivors in the central region from 1926 to 1965, although it has steadily recovered since then, apparently with little contribution of immigrants from outside Scandinavia (Linnell, Broseth, Odden, & Nilsen, 2010). In NE Poland the population became restricted to the Białowieża Primeval Forest (BPF), with apparent absence of lynx from 1890 to 1914 (Bieniek, Wolsan, & Okarma, 1998; Jędrzejewski et al., 1996), followed by a short bottleneck during the 1960s and 1970s and a modest recovery assisted by immigrants by the end of the 20th century (Jędrzejewski et al., 1996). In contrast, the Carpathians population has been considered relatively large, although largely isolated from other lynx populations, and has been used as the source of animals for reintroductions in central/western Europe (Von Arx, Breitenmoser‐Würsten, Zimmermann, & Breitenmoser, 2004). However, this population has not been exempted from intense direct persecution that left around 100 individuals in Romania by 1930 (Kratochvil, 1968), and probably similar numbers in the Slovakian part (Hell & Slamečka, 1996). The protection of the species in both countries in the 1930s allowed a significant recovery of the population with around 500 individuals in Romania by 1950 and 400–500 in Slovakia in the period 1960–1990 (Kubala et al., 2019). Similar scenarios with western fragmented and bottlenecked populations due to continued human pressure during the Holocene versus more contiguous and stable ranges in the east have also been postulated for other carnivore species such as wolf and brown bear in Europe (Adamec et al., 2012; Hindrikson et al., 2017; Pilot et al., 2014).

### 4.3 Implications for conservation, management and taxonomy

For conservation and taxonomic purposes, it is critical to formally assess possible adaptive intraspecific divergences within the Eurasian lynx, a possibility that is now made feasible by the availability of genomic data. Even though we find little to no support for most of the subspecies discussed in the literature in recent years, the finding of shallow differentiation at neutral regions of the genome does not exclude the occurrence of locally divergent selection at particular genes (i.e., local adaptation). In addition, we cannot discard the possibility that the morphological variation that sustained previous subspecific delimitations represents plastic responses to local environments mediated by epigenetic changes, as suggested by a recent study of *Lynx canadensis* (Meröndun et al., 2019). Nevertheless, given our results, we argue that management plans should focus on reversing the demographic trends to prevent further genetic erosion in the most affected populations, and allowing natural evolutionary processes, including the facilitation of population connectivity through migratory corridors in human‐altered habitats, as already postulated for lynx and other large carnivores in Europe (Boitani et al., 2015). Management based on maintaining the current distinctiveness of endangered European populations may not be warranted given the shared history and sustained historical gene flow inferred in this study, and should be balanced against risks of inbreeding depression, which in the absence of further conclusive evidence are likely to exceed those of outbreeding depression in these populations (Frankham, 2015; Frankham et al., 2011).

## AUTHOR CONTRIBUTIONS

K.S., J.A.G. and M.R. conceived the project and designed the study; A.P.S., G.N., I.O., I.V.S., J.A.G., K.S., M.G.D. and M.P. provided samples; E.M., K.W. and M.L.P. performed the laboratory work; B.M.C., D.K.R., E.M., K.W. and M.L.P. analysed the data; B.M.C., D.K.R., E.M., J.A.G., K.S., M.L.P. and M.R. interpreted the results; M.L.P. drafted the manuscript with support from B.M.C., D.K.R., E.M. and J.A.G. and critical input from A.P.S., K.S. and M.R; J.A.G. supervised the project. All authors approved the final version of the manuscript.

## OPEN RESEARCH

Bam files generated in this study have been deposited in the European Nucleotide Archive (study accession no. PRJEB28038). Mitogenomic consensus sequences for each sample have been deposited in GenBank (accession nos. MK229198–MK229293). Scripts used for bioinformatics analyses are available in: https://github.com/mlucenaperez/contemporary_analysis

## Data Availability

Raw sequence data are available in the European nucleotide archive (ENA) under the study primary accession code PRJEB28038. Mitochondrial haplotypes are available in GenBank under accession numbers: MK229198–MK229293.
