# Supplementary material for: Genomic patterns in the widespread Eurasian lynx shaped by Late Quaternary climatic fluctuations and anthropogenic impacts
Source: Mol Ecol. 2020 Feb 23;29(4):812–28. doi: 10.1111/mec.15366 (PMC7064982; doi:10.1111/mec.15366)
Supplement: Supplementary file 2 [file MEC-29-812-s002.pdf]

MOLECULAR ECOLOGY

Supplemental Information for:

Genomic patterns in the widespread Eurasian lynx shaped by Late Quaternary climatic fluctuations and anthropogenic impacts

Maria Lucena-Perez[a], Elena Marmesat[a], Daniel Kleinman-Ruiz[a], Begoña Martínez-Cruz[b], Karolina Węcek[c], Alexander P. Saveljev[d], Ivan V. Seryodkin[e], Innokentiy Okhlopkov[f], Mikhail G. Dvornikov[g], Janis Ozolins[h], Galsandorj Naranbaatar[i], Milan Paunovic[j], Mirosław Ratkiewicz[k], Krzysztof Schmidt[c], José A. Godoy[a]*

[a] Department of Integrative Ecology, Estación Biológica de Doñana (CSIC), C/ Américo Vespucio 26, 41092 Seville, Spain
[b] School of Biological and Environmental Sciences, Liverpool John Moores University, Byrom Street, Liverpool, L3 3AF, UK
[c] Mammal Research Institute, Polish Academy of Sciences, 17-230 Białowieża, Poland
[d] Department of Animal Ecology, Russian Research Institute of Game Management and Fur Farming, 79 Preobrazhenskaya Str, Kirov, 610000, Russia; Biological Faculty of Moscow State University, Leninskie Gory 1/12, Moscow, 119234 Russia
[e] Laboratory of Ecology and Conservation of Animals, Pacific Institute of Geography of Far East Branch of Russian Academy of Sciences, 7 Radio Street, Vladivostok 690041, Russia.; Far Eastern Federal University, 8 Sukhanova Street, Vladivostok 690091, Russia
[f] Institute for Biological Problems of Cryolithozone, Siberian Division of the Russian Academy of Sciences, Yakutsk, Republic of Sakha, Russian Federation
[g] Department of Hunting Resources, Russian Research Institute of Game Management and Fur Farming, 79 Preobrazhenskaya Str, Kirov 610000, Russia
[h] Department of Hunting and Wildlife Management, Latvijas Valsts mežzinātnes institūts "Silava", Rīgas iela 111, Salaspils, Latvia
[i] Institute of General and Experimental Biology, Mongolian Academy of Science, Enkhtaivan avenue 54B, Ulaanbaatar-13330, Mongolia
[j] Natural History Museum, Njegoševa 51, 11111 Belgrade, Serbia
[k] Institute of Biology, University of Białystok, Ciołkowskiego 1 J, 15-245 Białystok, Poland

*Corresponding author: José A. Godoy; E-mail: godoy@ebd.csic.es.

Table of Contents:

SUPPLEMENTARY METHODS 4

History and status of the sampled populations 4

Samples 6

Genome re-sequencing 6

Neutral regions definition and exclusion 11

Mitogenome reconstruction 11

Treatment of suboptimal samples 12

Identification of the ancestral state 14

Chromosome X and Y regions definition and molecular sexing 14

Y chromosome variation 17

SNP calling and genotyping 18

Phylogenetic analyses of mitogenomes 18

SUPPLEMENTARY FIGURES 20

Fig. S1. PSMC results with bootstrapping 20

Fig. S2. Population trees inferred by Treemix when allowing zero, one, three, four, five and six migration events. 21

Fig. S3. Visualization of the residuals from the fit of the model to the data for m=2 in TreeMix 22

Fig. S4. Treemix results for 10 runs allowing two migration events. 23

Fig. S5. Fraction of variance explained by the model allowing 0 to 6 migration events in Treemix. 24

Fig. S6. Alternative clustering patterns obtained in 10 runs of NGSadmix for K=2, Mongolia population comprise K/C-aymag (i.e. Central and Khentii aymag) and Ömnögovi 25

Fig. S7. Correlation of genetic vs. geographic distances between pairs of individuals. 26

Fig. S8. NJ-tree of populations based on pairwise FST 27

Fig. S9. Alternative clustering patterns obtained in 10 runs of NGSadmix for K=3 28

Fig. S10. Alternative clustering patterns obtained in 10 runs of NGSadmix for K=4 29

Fig. S11. Alternative clustering patterns obtained in 10 runs of NGSadmix for K=5 30

Fig. S12. Alternative clustering patterns obtained in 10 runs of NGSadmix for K=6 31

Fig. S13. Procrustes-transformed PCA coordinates to geographical coordinates in European populations, and sampling sites 32

Fig. S14. Procrustes-transformed PCA coordinates to geographical coordinates in Asian populations, and sampling sites 33

Fig. S15. Correlation of genetic vs. geographic distances between pairs of individuals from European populations, including and excluding individuals from European bottlenecked populations 34

Fig. S16. Comparison of genomic nucleotide diversity among mammals 35

Fig. S17. Nucleotide diversity (π) values for autosomal sites and ratios of π diversity in Xchr vs. autosomes 36

Fig. S18. Site frequency spectrum (SFS) of the different Eurasian lynx populations.......... 37

Fig. S19. Comparison of mitogenomic pairwise differences (k) among different species.. 38

Fig. S20. Mitogenome haplotype network 39

REFERENCES 40

Supplementary Methods

History and status of the sampled populations

Information on the recent and current demographic status of Eurasian lynx populations is large lacking and mostly unreliable, being mostly based on unvalidated expert opinion and hunting statistics.   Nevertheless, the populations sampled do cover a range of recent demographic histories and exposure to anthropogenic impacts (Table S2). Westernmost populations in Europe are the remnants of an ancient range that started its contraction as early as the 7th century when the species disappeared from Great Britain and followed by a progressive eastward withdrawal in continental Europe starting probably in the 16th century and peaking in the 19th and 20th century with the implementation of extermination policies in many countries. Such intense persecution led to the extermination of the species in most of western Europe, with the few westernmost populations surviving going through intense and extended bottlenecks which were followed by a slow recovery once the species became protected in the 1930s-1940s. Bottlenecks and genetic isolation have been particularly intense in the Balkans, Norway (together with neighbouring Sweden) and NE-Poland, which were at the brink of extirpation during the first half of 20th century and with little or no gene flow from other lynx populations. The Carpathians Mountains (mainly Romanian and Slovakian parts) went also through important contractions leaving 100-200 individuals by the 1930s, but recovered to more than 1000 individuals by the second half of the 20th century. The Carpathian population has been since considered relatively large, but largely isolated from other lynx populations, and has been used as the source of animals for reintroductions in western Europe (Von Arx, Breitenmoser-Würsten,

Zimmermann, & Breitenmoser, 2004). However, the first model-based estimates obtained recently suggest lynx abundance in the region has been overestimated in the past (Krojerová-Prokešová et al., 2018; Kubala et al., 2017).

Latvia is part of the northwestern population, together with Finland, western Russia, and Estonia. While it also went through declines starting in the 17th century and intensifying in the early 20th century, the population recovered and now occupies its full historical range with 600-800 individuals estimated at present. Importantly, the population remained well connected to the large and continuous core population farther east in European Russia, which is represented in our sampling by Kirov and the Urals.

Information on the lynx populations in Asia is even more scarce, scattered and mostly consists of fur harvest data, which not only reflect population numbers but also socio-economic circumstances. The Asian distribution is generally considered continuous and with high densities, although these typically fluctuate due to natural (prey availability) and anthropic causes (harvest pressure), with amplitudes ranging from 7 to 13 years, being larger at northern latitudes (Matyushkin & Vaisfeld, 2003). Asian lynxes underwent also a heavy exploitation during last centuries, dating back to the XVII century, with 10 000 lynx pelts were recorder yearly in Siberia only (Brass, 1911). This, however, did not lead to the extirpation of entire populations typical for the bottlenecked populations in the western peripheries of the species range.

Samples

We sampled 80 *L. lynx* across the distribution range of the species, including individuals for five out of the six subspecies proposed by the IUCN Cat Specialist Group (*L. l. lynx, L. l. balcanicus, L. l. carpathicus, L. l. isabellinus,* and *L. l. wrangeli*) (Kitchener et al., 2018)). Majority of samples were tissues collected from legally hunted individuals (Norway, Latvia, Russia, and Romania; n = 68) or animals found dead (Poland, Carpathian Mountains, and Mongolia; n = 19). Three museum specimens (Balkans) and six blood samples of live-trapped lynx (Białowieża Primeval Forest in NE Poland and Carpathians Mountains, in Poland) were also obtained. Sampling of harvested individuals was opportunistic – the lynx harvesting was not intended for obtaining genetic samples. The license for lynx live-trapping and blood sampling in Poland was obtained from the National Ethics Committee for Animal Experiments (no: DB/KKE/PL – 110/2001) and the Local Ethics Committee for Animal Experiments at the Medical University of Białystok (no: 52/2007). These licenses adhere to the Directive 2010/63/EU on the protection of animals used for scientific purpose. No animals were harmed during live-trapping and handling.

Genome re-sequencing

Samples were sequenced at Centro Nacional de Análisis Genómico (CNAG-CRG) or Macrogen using different sequencing strategies (Table S1). These strategies depend on the quality of the gDNA extracted or the goal depth.

Library preparation

Library preparation 1. Good quality samples aimed to be sequenced at low to medium depth. gDNA samples were used for preparing Illumina sequencing compatible paired-end libraries using NO-PCR protocol, TruSeq™DNA Sample Preparation Kit v2 (Illumina Inc.) and the KAPA Library Preparation kit (Kapa Biosystems). In short, 2.0 micrograms of genomic DNA sheared on a Covaris™ E210 was end-repaired, adenylated and ligated to Illumina specific indexed paired-end adaptors. The DNA was size selected with AMPure XP beads (Agencourt, Beckman Coulter) in order to reach the fragment insert size of 220-550bp. The final libraries were quantified using Library Quantification Kit (Kapa Biosystems).

Library preparation 2. Low input gDNA samples aim to be sequenced at low to medium depth. Low input gDNA was used for short-insert paired-end libraries for whole genome sequencing were prepared with KAPA HyperPrep kit (Roche-Kapa Biosystems) with some modifications. In short, 0.8-1.0 microgram of genomic DNA was sheared on a Covaris™ LE220 (Covaris) in order to reach the fragment size of ~500bp. The fragmented DNA was further size-selected for the fragment size of 220-550bp with AMPure XP beads (Agencourt, Beckman Coulter). The size selected genomic DNA fragments were end-repaired, adenylated and ligated to Illumina sequencing compatible indexed paired-end adaptors (NEXTflex® DNA Barcodes). The adaptor-modified end library was size selected and purified with AMPure XP beads to eliminate any not ligated adapters. The final libraries were quantified using Library Quantification Kit (Kapa Biosystems).

Library preparation 3. Good quality samples aim to be sequenced at medium to high depth. Library preparations were performed according to TruSeq DNA PCR-Free library

prep guide (Illumina). Briefly, one microgram of quantified genomic DNA was sheared using an LE220Focused-ultrasonicator (Covaris, Inc.) with a Duty factor of 15%, Peak incident power 450 W, 200 cycles per burst for 50 seconds. Sheared DNA fragments were end-repaired, size-selected to obtain DNA fragments around 350 bp, and adenylated according to the manufacturer's instructions. After ligating indexing adapters to the ends of the DNA fragments, quality and band size of libraries were assessed using D1000 Screen Tapes (Agilent) on a TapeStation 2200 (Agilent). Library concentration was measured by qPCR using KAPA library Quantification Kit (KAPA Biosystems).

Library preparation 4: Low input gDNA samples aim to be sequenced at medium to high depth. Library preparations were performed according to TruSeq Nano DNA library prep guide (Illumina). Briefly, 100 nanogram of quantified genomic DNA was sheared using an LE220Focused-ultrasonicator (Covaris, Inc.) with a Duty factor of 15%, Peak incident power 450 W, 200 cycles per burst for 50 seconds. Sheared DNA fragments were end-repaired, size-selected to obtain DNA fragments around 350 bp, and adenylated according to the manufacturer's instructions. After ligating indexing adapters to the ends of the DNA fragments, DNA libraries were enriched using eight cycles of PCR according to the manufacturer's instructions. Quality and band size of libraries were assessed using D1000 Screen Tapes (Agilent) on a TapeStation 2200 (Agilent) after size selection and after PCR amplification. Libraries were quantified by qPCR using KAPA library Quantification Kit (KAPA Biosystems).

Clustering and Sequencing

HiSeq2000 v3, 2x101bp. Some libraries were sequenced using TruSeq SBS Kit v3-HS (Illumina Inc.), in paired end mode, 2x101bp, in a fraction of a sequencing lane of HiSeq2000 flowcell v3 (Illumina Inc.) according to standard Illumina operation procedures. Primary data analysis, the image analysis, base calling and quality scoring of the run, were processed following standard Illumina procedures.

HiSeq2000 v4, 2x126bp. Other libraries were sequenced on HiSeq2000 (Illumina, Inc) in paired-end mode with a read length of 2x126bp using TruSeq SBS Kit v4 following the manufacturer's protocol. Image analysis, base calling and quality scoring of the run were processed following standard Illumina procedures.

HiSeq X-10, 2x150bp. Libraries aim to be sequenced at medium to high depth were sequenced on HiSeq X-10 sequencer (Illumina, Inc) in paired-end mode with a read length of 2x150bp following the manufacturer's protocol. Image analysis, base calling and quality scoring of the run were processed following standard Illumina procedures.

*Library preparation 1 and 2 and Sequencing using HiSeq2000 was carried out in CNAG facilities. Library preparation 3 and 4 and Sequencing using HiSeqX-10 was carried out in MACROGEN facilities.

In all cases, samples were sequenced using Illumina protocols, and primary data analysis was carried out with the standard Illumina pipeline.

Quality controls, trimming & mapping to nuclear genome

We evaluated the quality of the data using FastQC (https://www.bioinformatics.babraham.ac.uk/projects/fastqc) and removed adaptors using SeqPrep (https://github.com/jstjohn/SeqPrep). Trimmed reads were mapped to a 2.4Gb *Lynx pardinus* (Iberian lynx) reference genome (Abascal et al., 2016) using BWA-MEM (Li, 2013) with default parameters. After adding read groups to each sample using picard-tools (https://broadinstitute.github.io/picard/), we merged the bam files from the same individuals, which are also coming from the same library, with SAMtools merge (Li et al., 2009). We marked the duplicates using picard-tools (https://broadinstitute.github.io/picard/) and performed a local realignment using GATK 3.4 (McKenna et al., 2010). Then, we performed a Base Quality Score Recalibration (BQSC), a pre-processing step to detect systematic errors made by the sequencer. As BQSC needs a set of known variants, which we did not have, we followed GATK recommendations (https://gatkforums.broadinstitute.org/gatk/discussion/44/base-quality-score-recalibration-bqsr), and did an initial round of SNP calling on our data unrecalibrated, and used it afterwards to do BQSR. Variant calling was done using HaplotypeCaller (McKenna et al., 2010) on *a priori* defined populations (considered as sampling locations, see Table S1, Table S2). We filtered the resulting VCF files (https://github.com/vcflib/vcflib, ”QD > 2 & MQ > 40 & FS < 100”), and used those VCF file only to perform BQSC of the data using GATK 3.4 (McKenna et al., 2010). We calculated overall stats using SAMtools flagstat (Li et al., 2009) and average depth using SAMtools depth (Li et al., 2009). The depth of our samples was on average 7.4

+/- 2.3 sd for those sequenced using HiSeq 2000 and 23.7+/- 3.7 sd for those sequenced with HiSeq X-10.

Neutral regions definition and exclusion

To determine neutral regions of the nuclear genome we excluded all the regions annotated as functional categories in the Iberian lynx reference genome, i.e. genes, lncRNAs, mRNAs, and ncRNAs (Abascal et al., 2016). Besides, we annotated and excluded promoters of protein-coding genes and lncRNA defined as 1000-bp upstream of the gene or lncRNA. We annotated and excluded ultra conserved non-coding elements (UCNEs) using human coordinates (https://ccg.vital-it.ch/UCNEbase/). This information was translated into cat coordinates using LiftOver (http://rohsdb.cmb.usc.edu/GBshape/cgi-bin/hgLiftOver), and latter, we translated these cat coordinates into lynx coordinates by using lynx to cat sinteny (Abascal, 2016). We also added a security buffer of +/-1000bp to any functional region to avoid regions under the influence of functional areas. To exclude these functional regions and our security buffer from the bam files we used bedtools subtract and bedtools intersect (Quinlan & Hall, 2010).

Mitogenome reconstruction

To reconstruct the mitogenome, we mapped only 10M random reads in the case of WG samples while all reads coming from the capture experiment were used. The average depth was calculated using Samtools depth piped to a custom bash script.

We called SNPs using the following settings on Freebayes (Garrison & Marth, 2012): haploid genome, minimum read mapping quality 30, minimum base quality 20, a minimum of two bases supporting an alternative allele call, and a maximum read mismatch fraction of 0.2. Repetitive regions RS2 and RS3 (positions 16096-16382 and 16908-174, respectively) (Sindičić et al., 2012), where no SNPs could be called reliably, were excluded from the analysis. The number of reference positions not covered by any read was calculated for each individual using the command genomecov in BEDTools (Quinlan & Hall, 2010). A consensus for each mitochondrial genome was constructed using the FastaAlternateReferenceMaker command in GATK (McKenna et al., 2010). Consensus sequences were rearranged to present them with standard coordinates but lacking the excluded repetitive regions.

Treatment of suboptimal samples

Balkans samples were museum material with unknown collection date. Some evidence indicates that at least two of them were more than 70 years old. All three samples were maintained in suboptimal conditions for DNA studies. Accordingly, we processed the samples in a sterile lab especially used for museum or suboptimal material. DNA extraction yielded relatively low amount of DNA and the DNA was highly degraded. Still, given the interest of the samples, we proceeded with library preparation. Libraries failed standard quality control because of degradation and RNA contamination. One of them also failed in DNA quantity. Standard procedures were implemented to prevent contamination in the lab throughout the whole process. As they were suboptimal material they underwent two sequencing runs to reach our goal depth, in contrast with the rest of the samples that got this depth with only one run.

FASTQC analysis indicates that the reads show a distorted GC, which could be an indication of bacterial DNA. We mapped the reads to the nuclear genome, and calculated the endogenous content as: reads mapped *vs.* total reads. Balkans samples showed 67%, 73%, and 85%, way below the average for the rest of the samples (~97%). We also calculate Ts/Tv ratio. High Ts/Tv ratios are indication of damaged DNA, and are typical of museum and ancient materials. While one of the samples (labeled as historical) showed signs of damage when compared to the rest of contemporary samples, the other two showed a very low value. We performed diversity measures using ANGSD and SNP calling using GATK and we found unexpectedly high diversity values (above twice the diversity of Tuva (the population with the highest diversity). Therefore, we decide to exclude these samples from this analysis. Still, we think that analysis such as structure or PCA, although taken with caution, could give some useful information on the relationship of Balkan samples to the rest of the samples. Contamination, errors, and damage are unlikely to be shared among samples, so the variants shared by all three samples and separating Balkan samples from the rest are likely to be authentic.

For the mitogenome analysis, Balkans samples underwent a BLAST+ (Camacho et al., 2009) based filtering step between mapping and SNP calling. BLAST+ searches were performed in our own server against NCBI database using as queries all mapping reads showing one or more mismatches to the reference genome. Reads were excluded if the list of hits with the lowest E-values did not include at least one felid species. Besides, we manually revised and confirmed all SNPs called from these samples. Therefore, the extra divergence of the Balkan mitogenomes, obtained from low quality

samples, is not likely caused by contamination or post-mortem damage, as many divergent reads potentially coming from exogenous DNA were efficiently removed through this BLAST filtering step (sample:percentage of removed reads, mapping reads retained post-filtering: h_ll_ba_0214: 34%, 18370; h_ll_ba_0215: 17%, 26622; c_ll_ba_0216: 8%, 30749). Moreover, the exact same haplotype was independently reconstructed from the three samples; suggesting that they are not affected by contamination or damage, which are expected to be random and sample-specific.

Identification of the ancestral state

We inferred the ancestral state using *L. rufus* for our analysis with ANGSD. Raw paired-end Illumina reads were mapped to the Iberian lynx reference genome and the resulting bam file was used to call variants using SAMtools mpileup (–q30) (Li et al., 2009). The output was piped into pu2fa (-C45) program from Chrom-Compare-master (https://github.com/Paleogenomics/Chrom-Compare) to do a pseudo-haploidisation. We inferred the ancestral state for 97% of bases in our reference genome.

Chromosome X and Y regions definition and molecular sexing

The annotation of the reference genome do not include sexual chromosomes so we characterized such regions as they should be treated apart of the autosomes because of their different ploidy. For each sexual chromosome we defined two different sets of regions depending on their posterior use:

i) A lax definition was used to define any region having signals of belonging to a sex chromosome. We used these regions to sex molecularly our

individuals and to exclude them from any analysis intended on the autosomes (i.e. PSMC, measures of autosomal diversity).

ii) An strict definition was used to define regions which showed clear signs of belonging to the sexual chromosomes and therefore were used in the analysis aiming to study haploid and sex-linked genetic signals.

i) For our lax definition, we defined non-recombining parts of sexual chromosomes regions using differential depth expected in autosomal *vs* Y and non-recombinant X chromosome regions. Expecting that female/male ratio would be: ~1 in autosomal regions ~0 in Y non-recombinant regions ~0.5 in X non-recombinant regions. Similar approaches were previously used by Hall et al. (2013), and later modified by Smeds et al. (2015). We used *Lynx pardinus* individuals (eleven males and 15 females) sequenced to 5x depth to do this analysis. First because the sex of those individuals was known, and also, because our reference genome belongs to *Lynx pardinus* and only perfectly matching reads are taken into account to calculate read depth -species divergence could cause lack of depth in some areas of the genome. Then, we extrapolated our results to our *L. lynx* individuals, assuming that the divergence of the two species is so recent that chromosomal reorganizations, if any, should be a minority. We computed the per base average normalized depth for both females and males and then calculated the female/male ratio.

First, we used samtools view to select only primary alignments with a minimum mapping quality of 30 and no mismatches followed by samtools depth to get each base depth. Then, for each individual we used its mode to normalize each base depth to

later compute the average depth per base and sex which was used to get the per base female normalized depth / male normalized depth ratio. The distribution of such ratios was explored in R using a random subset of 5M positions.

We decided to make the definition (X, Y or autosomic) of each region using contigs as the meaningful units, as single bases belonging to a chromosome when the adjacent ones do not does not make biological sense and scaffolds might be chimerical due to errors in the scaffolding process. For each contig we obtained its length as well as the number of bases with no data available.

To further inform the definition of the X chromosome, we took advantage of the definition of the regions annotated as syntenic to cat's X or autosomal chromosomes in Abascal (2016). First, we compared the distributions of the ratios found in those syntenic regions in contrast with regions syntenic to autosomes to help us to define the thresholds used. A base was assigned to the X chromosome if it showed a female depth/male depth ratio above 1.5 and/or if it had been described as syntenic to the cat X chromosome. We defined a contig as belonging to the X chromosome in our lax definition if more than 30% of its bases met our requirements.

The lax definition of the Y chromosome contigs required them to have more than 30% of their bases with a female/male depth ratio below 0.3 and at least a normalized depth of 0.2 in males (to avoid non-covered regions by sequencing or mapping biases).

ii) As the strict X chromosome definition we simply used the bases that had already been described as X chromosome using the synteny to the cat genome.

As the strict definition, we required at least 90% of the total bases in a contig to have a ratio below 0.3 and an average normalized depth for males between 0.2 and 0.8 (to also exclude multicopy Y chromosome regions) for the Y chromosome.

Chromosome\Definition	Lax (scaffolds/bases)	Strict (scaffolds/bases)
Y Chromosome	401 / 335,035	77 / 33,032
X Chromosome	14,467 / 72,426,678	1,891 / 45,245,057

To sex molecularly our individuals, we called SNPs on each sample's original BAM file using the lax definition of the Y chromosome as target region using GATK 3.4 (McKenna et al., 2010) and following GATK best practice recommendations (DePristo et al., 2011). Then we visually inspected the VCF files to define as females those samples showing virtually no depth for any reported SNP compared to males, which showed consistent depth in all regions.

Y chromosome variation

We called SNPs on males exclusively using their pooled BAM files, selecting our strict definition of Y chromosome bases as the target region, and the same setting used for the mitogenome calling.

SNP calling and genotyping

When required for the analysis we called variants following the genome VCF (GVCF) workflow in GATK 3.4 (McKenna et al., 2010)), i.e. we ran the HaplotypeCaller tool individually on each *L. lynx* sample to produce per-sample intermediate GVCFs, which were parsed to the GenotypeGVCFs tool for a joint variant calling and genotyping. Next, INDELs and multiallelic SNPs were filtered out from the VCF, as were all other variants that did not meet the recommended GATK hard filters (variants were excluded if "QD < 2.0 || FS > 60.0 || MQ < 40.0 || MQRankSum < -12.5 || ReadPosRankSum < -8.0 || SOR > 3.0").

Phylogenetic analyses of mitogenomes

In order to estimate a substitution rate for the mitochondrial genome in felids, we downloaded from GenBank the sequences and the respective annotations of the following felid mitogenomes: *Panthera leo* (GenBank: KP202262), *P. pardus* (GenBank: KP202265), *P. onca* (GenBank: KP202264), *P. uncia* (GenBank: KP202269), *P. tigris* (GenBank: KP202268), *Neofelis nebulosa* (GenBank: KP202291), *Acinonyx jubatus* (GenBank: KP202271), *Puma concolor* (GenBank: KP202261), *Catopuma temminckii* (GenBank: KP202267), *L. lynx* (GenBank: KP202283), *Pardofelis marmorata* (GenBank: KP202263), *Felis catus* (GenBank: FCU20753), *Prionailurus bengalensis* (GenBank: KP246843), *Caracal caracal* (GenBank: KP202272), and *Leopardus guigna* (GenBank: KP202293), and *Prionodon pardicolor* (GenBank: NC_024569) as a non-felid outgroup. Sequences were aligned using ClustalW v2.1 in Geneious v11.1.2 (Kearse et al., 2012). We used the Greedy algorithm with linked branch lengths implemented in

PartitionFinder v2.1.1 (Lanfear, Frandsen, Wright, Senfeld, & Calcott, 2017) to investigate the set of partitions and substitution models adequate to all possible combinations of tRNAs (pulled together), the two rRNAs and twelve genes in the dataset of the 16 mitochondrial sequences, encompassing 14,994 bp. The ND6 gene and the hypervariable Control Region were excluded from the analyses. We inferred a substitution rate of $1{,}54*10^{-8}$ mutations/site/year for the mitogenome.

We performed a Bayesian phylogenetic analysis with the 15 feline species using BEAST v2.4.8 (Bouckaert et al., 2014) and the optimal partitions selected with PartitionFinder among the models implemented in BEAST. The tree was calibrated using a set of fossil constraints (Table below). We assumed a strict clock as justified before (Paijmans et al., 2017) and a calibrated Yule model as the tree prior, more appropriate when considering sequences from different species. The Markov chain Monte Carlo (MCMC) chain was run for 50 million steps to ensure convergence of the chain and ESS values well above 200 for all parameters estimated. Results were visualised with Tracer v1.7 (Beta version) (Rambaut, Drummond, Xie, Baele, & Suchard, 2018) discarding the initial 10% as burn-in.

			Log Normal Parameters	
Fossil (estimated age)	Minimum hard bound	Maximum soft bound	M	S
Lynx (5.3 Ma)	5.3	10	1.0	1.25
Acinonyx (3.8 Ma)	3.8	10	1.2	1.3
Caracal (3.8 Ma)	3.8	16	2.0	1.5
Oldest Panthera tigris (1.5 Ma)	1.5	10	1.5	1.35
Oldest Panthera (3.8 Ma)	3.8	16	2.0	1.5

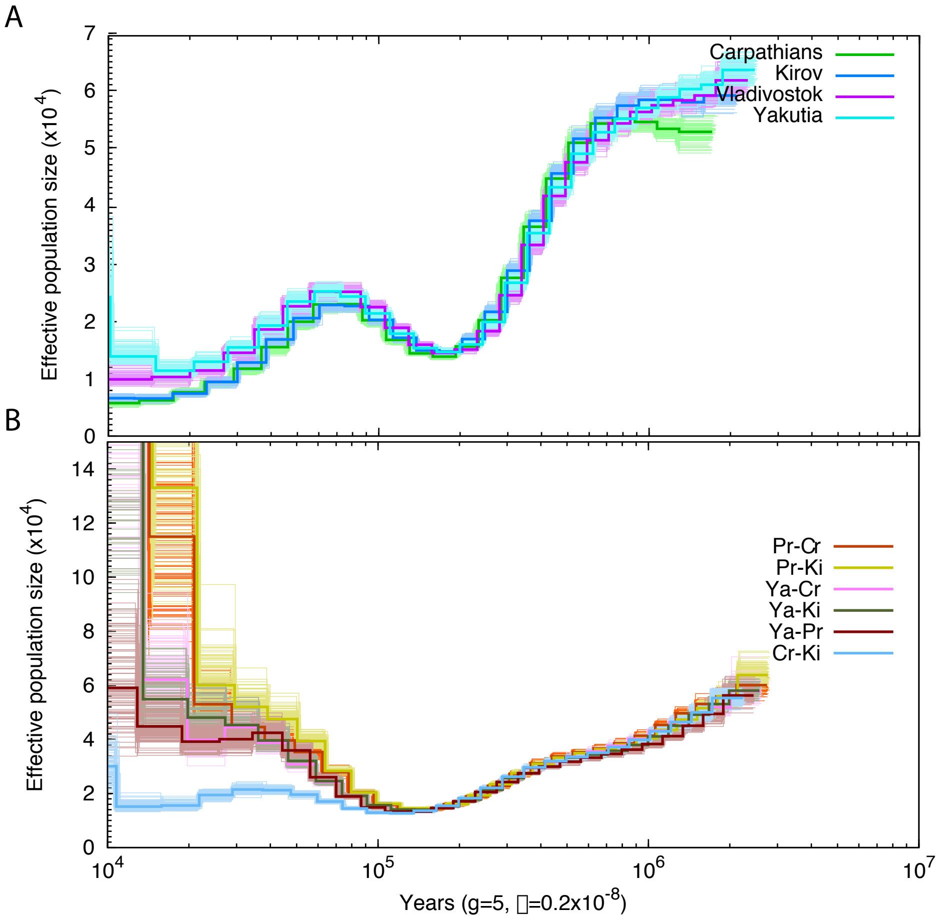

Fig. S1. PSMC results with bootstrapping for individuals from Kirov, Yakutia, Primorsky Krai and Carpathians, and for pseudo-diploid sequences created by combining haplotypes from pairs of populations.

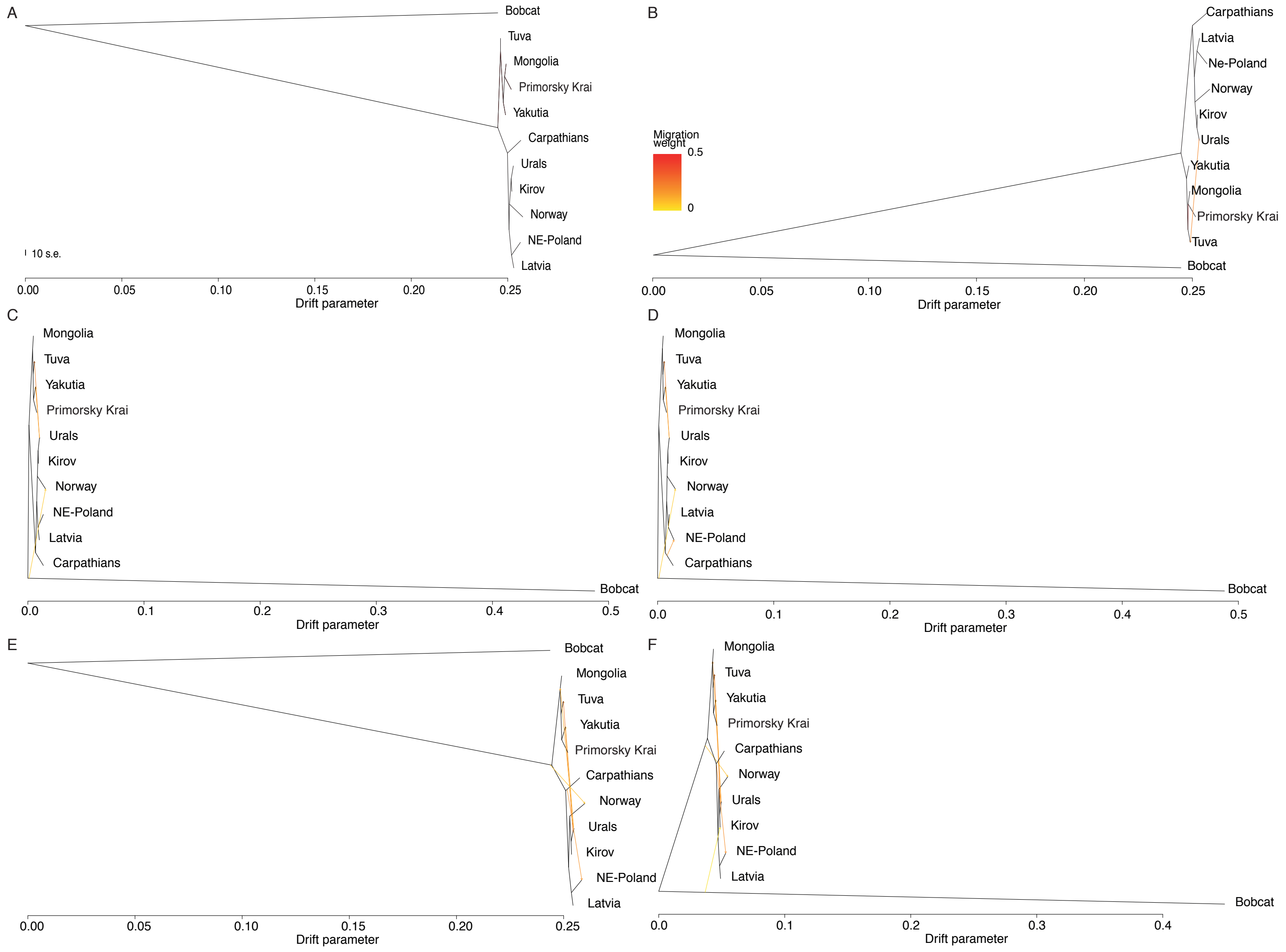

Fig. S2. Population trees inferred by Treemix when allowing zero, one, three, four, five and six migration events.

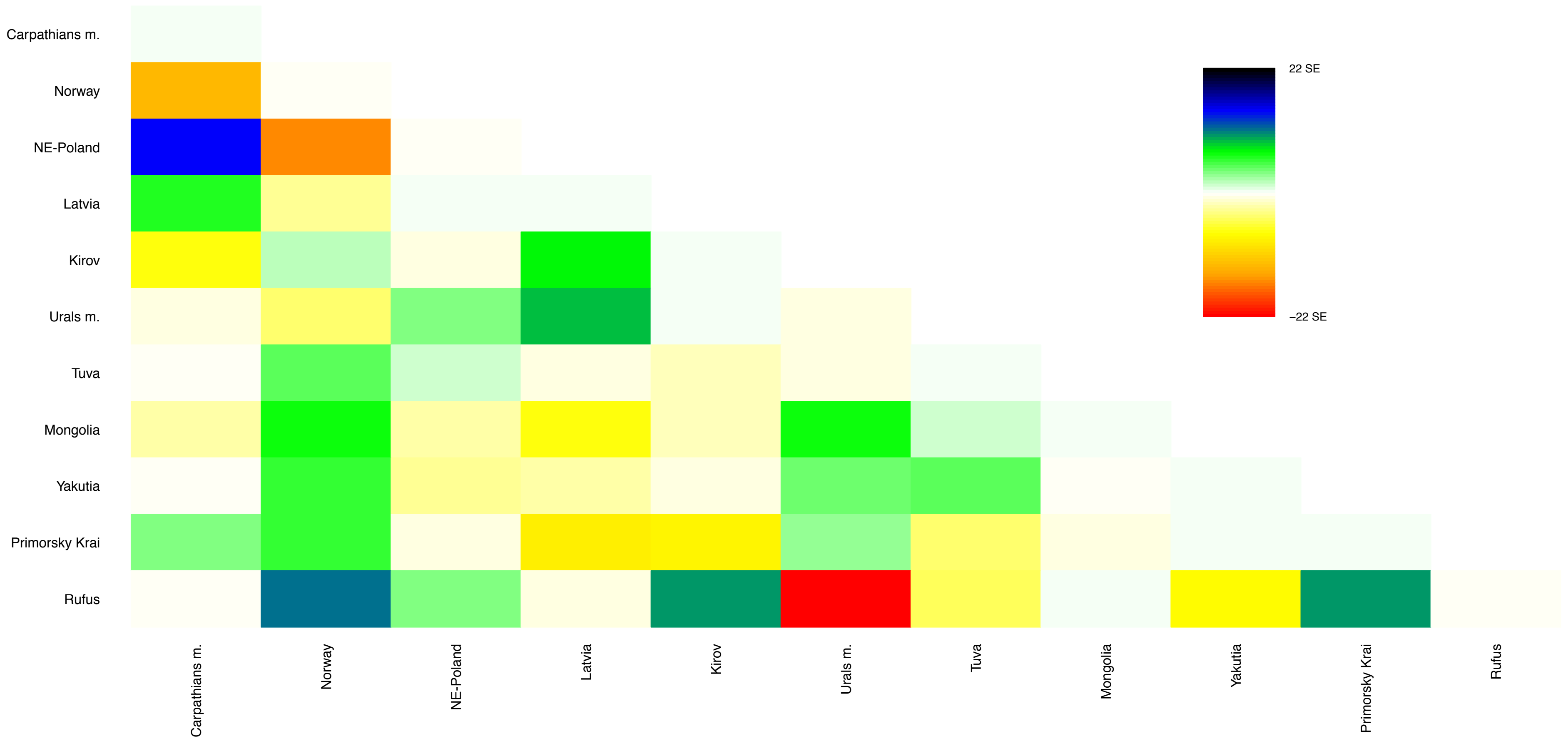

Fig. S3. Visualization of the residuals from the fit of the model to the data for m=2 in TreeMix.

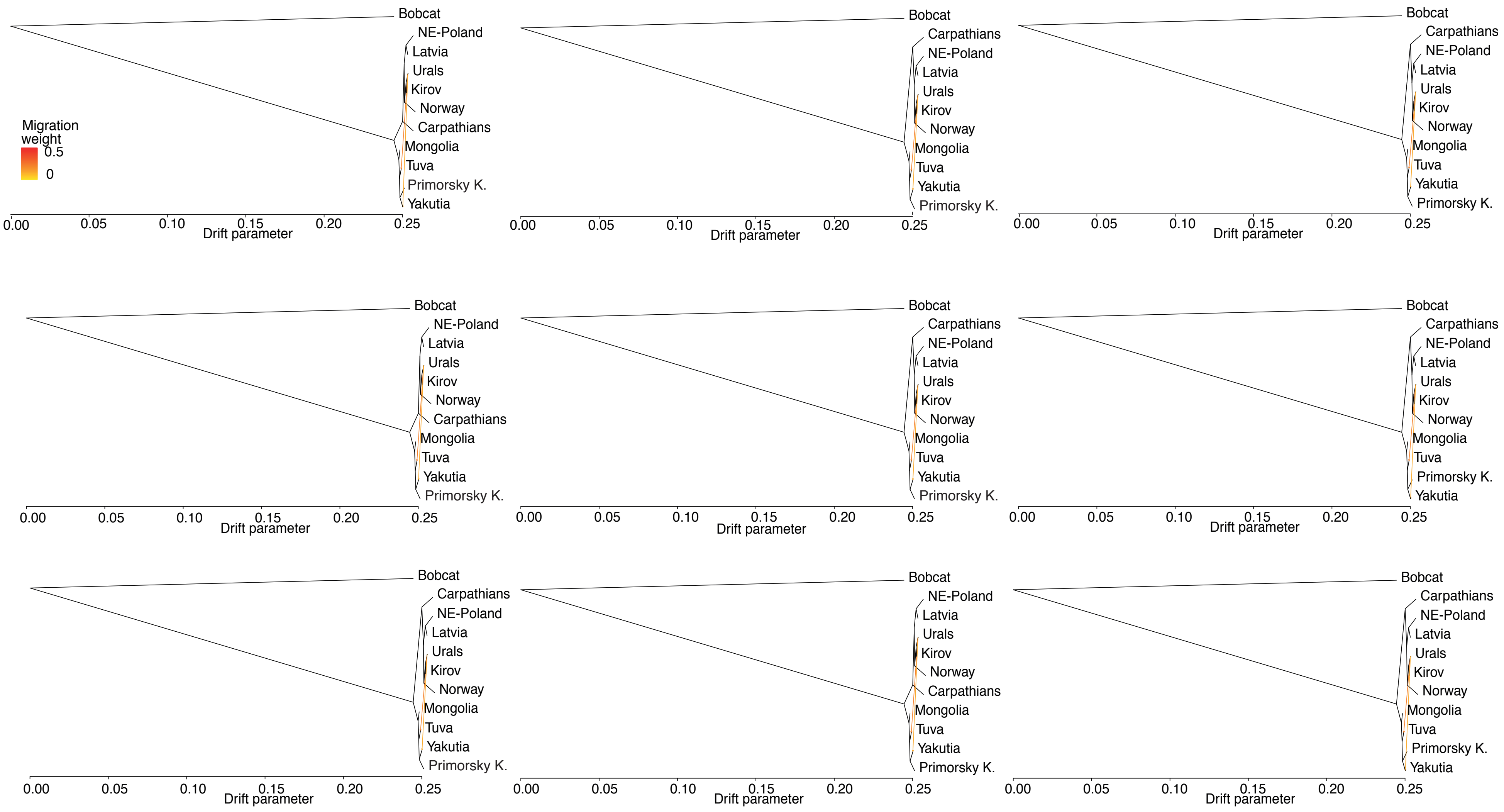

Fig. S4. Treemix results for 10 runs allowing two migration events.

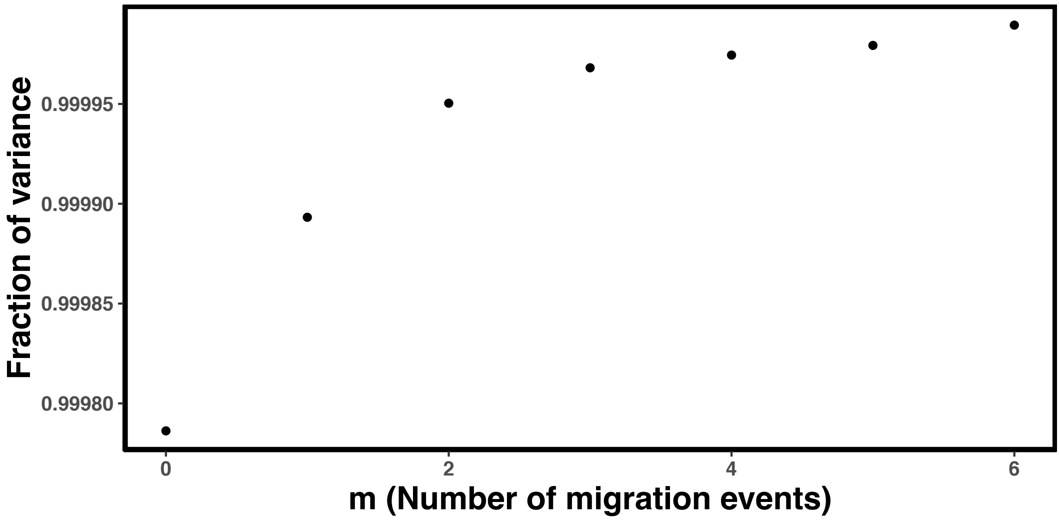

Fig. S5. Fraction of variance explained by the model allowing 0 to 6 migration events in Treemix.

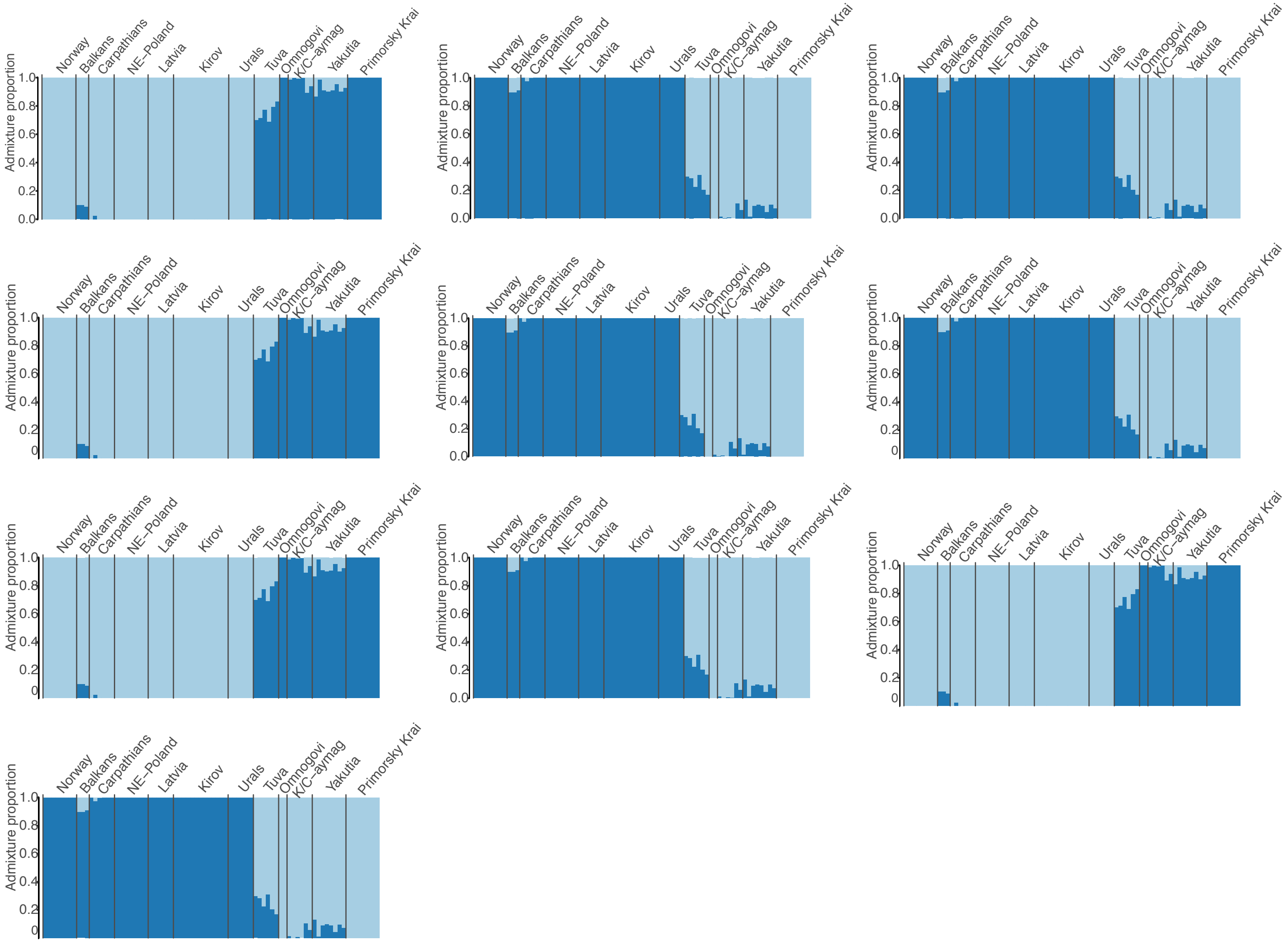

Fig. S6. Alternative clustering patterns obtained in 10 runs of NGSadmix for K=2, Mongolia population comprise K/C-aymag (i.e. Central and Khentii aymag) and Ömnögovi.

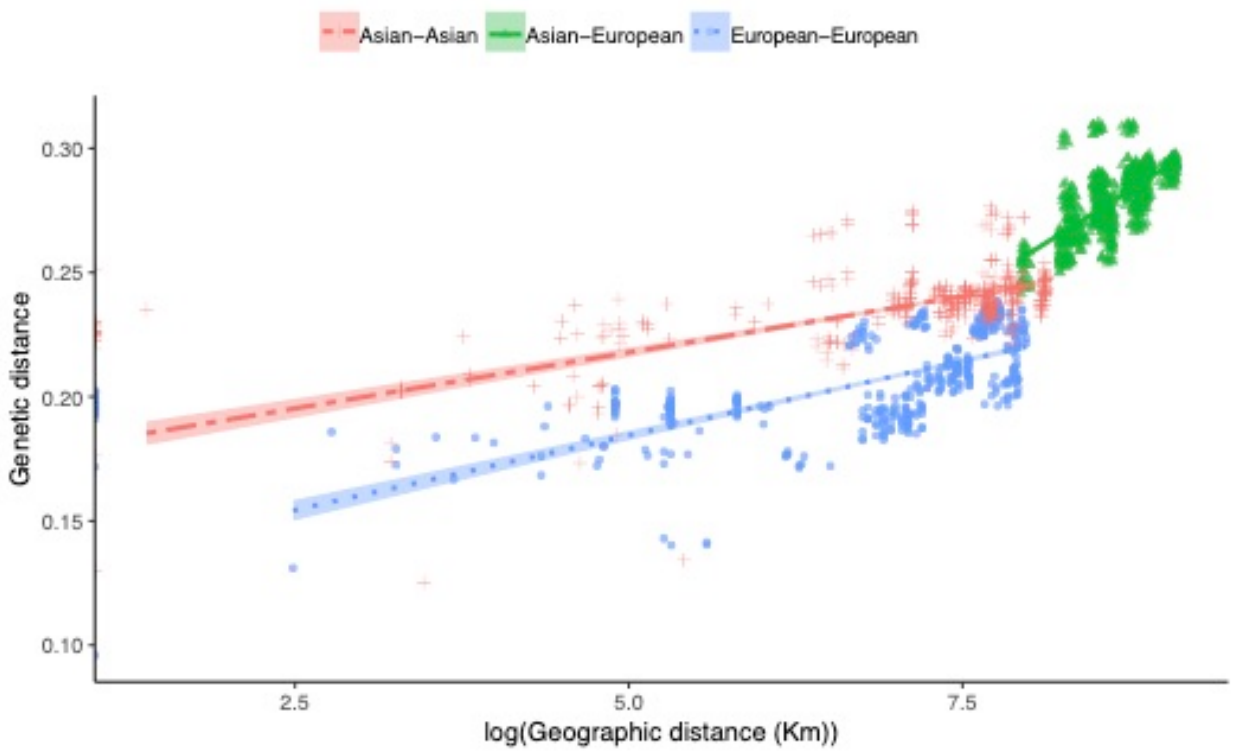

Fig. S7. Correlation of genetic vs. geographic distances between pairs of individuals. Correlations were assessed separately for Asian-Asian, European-European and Asian-European pairs. European bottlenecked populations (Carpathians, Norway and NE-Poland) were not included in this analysis. We tested an effect additional to geographic distance contributing to the genetic distance separating Asian and European individuals through a partial Mantel test. This test was significant (r=0.5459; p=0.001).

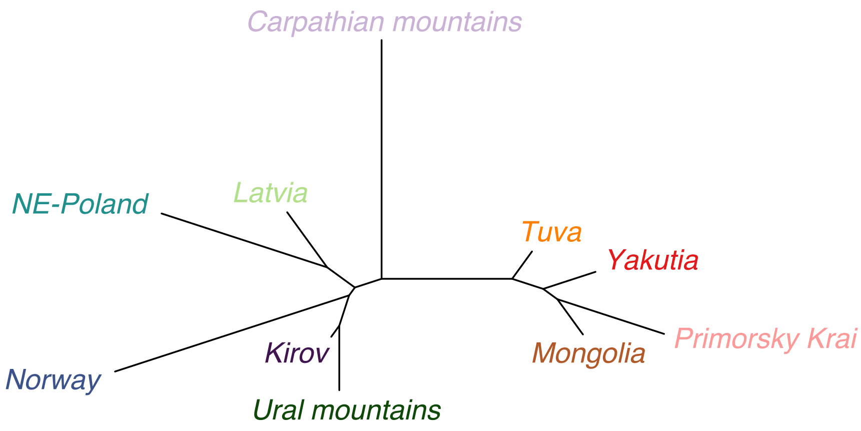

Fig. S8. NJ-tree of populations based on pairwise FST.

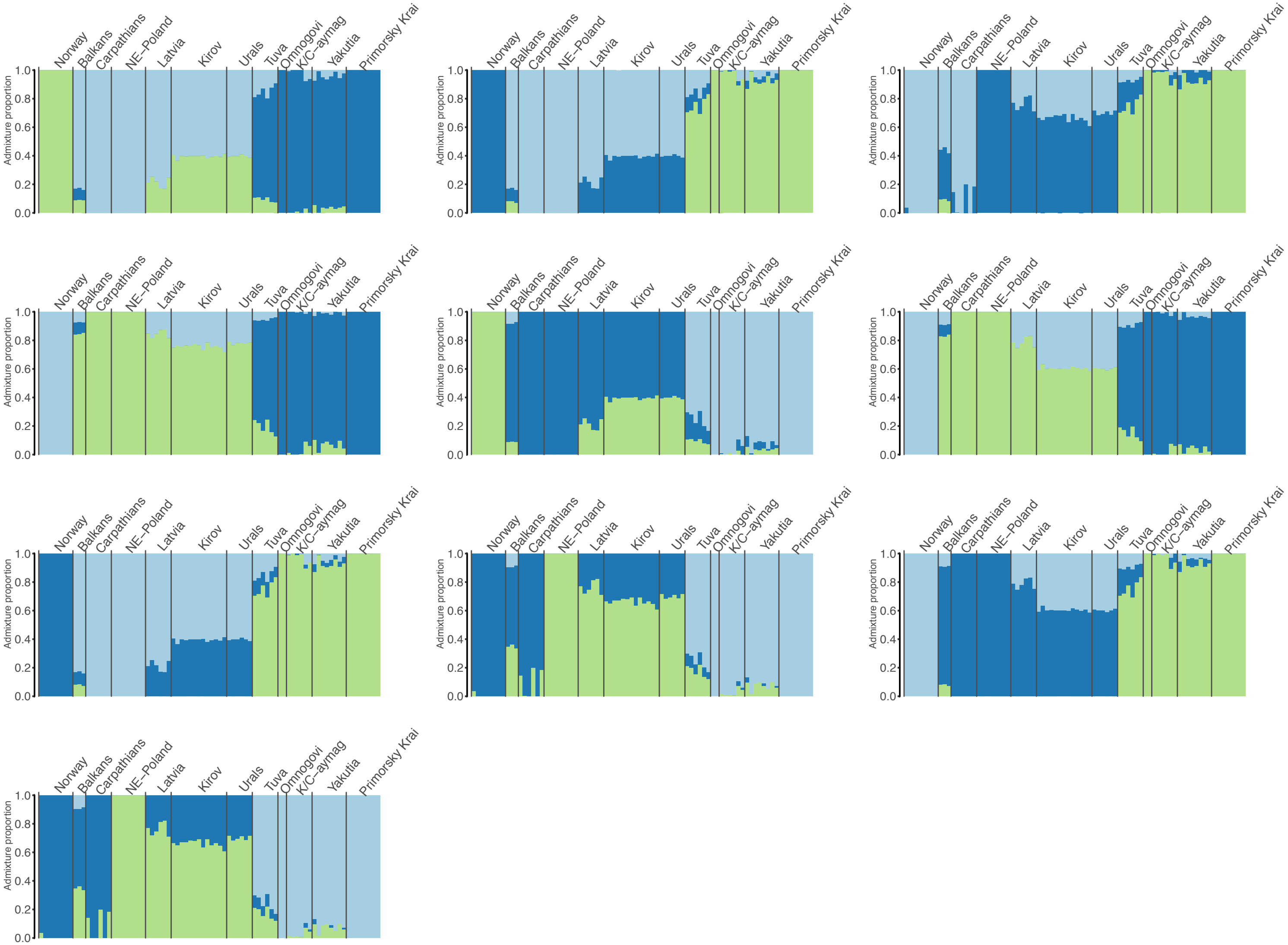

Fig. S9. Alternative clustering patterns obtained in 10 runs of NGSadmix for K=3. Mongolia population comprise K/C-aymag (i.e. Central and Khentii aymag) and Ömnögovi.

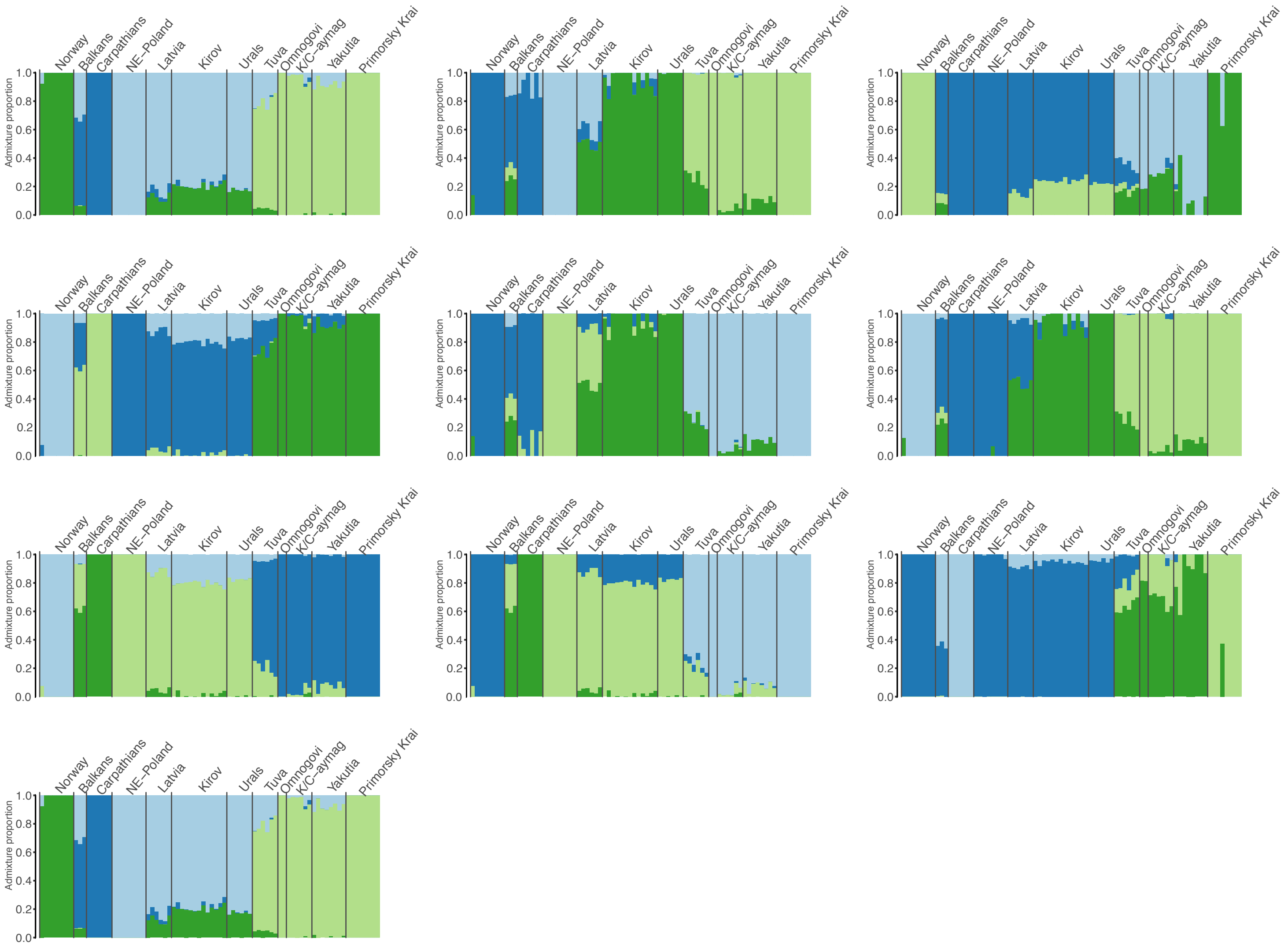

Fig. S10. Alternative clustering patterns obtained in 10 runs of NGSadmix for K=4. Mongolia population comprise K/C-aymag (i.e. Central and Khentii aymag) and Ömnögovi.

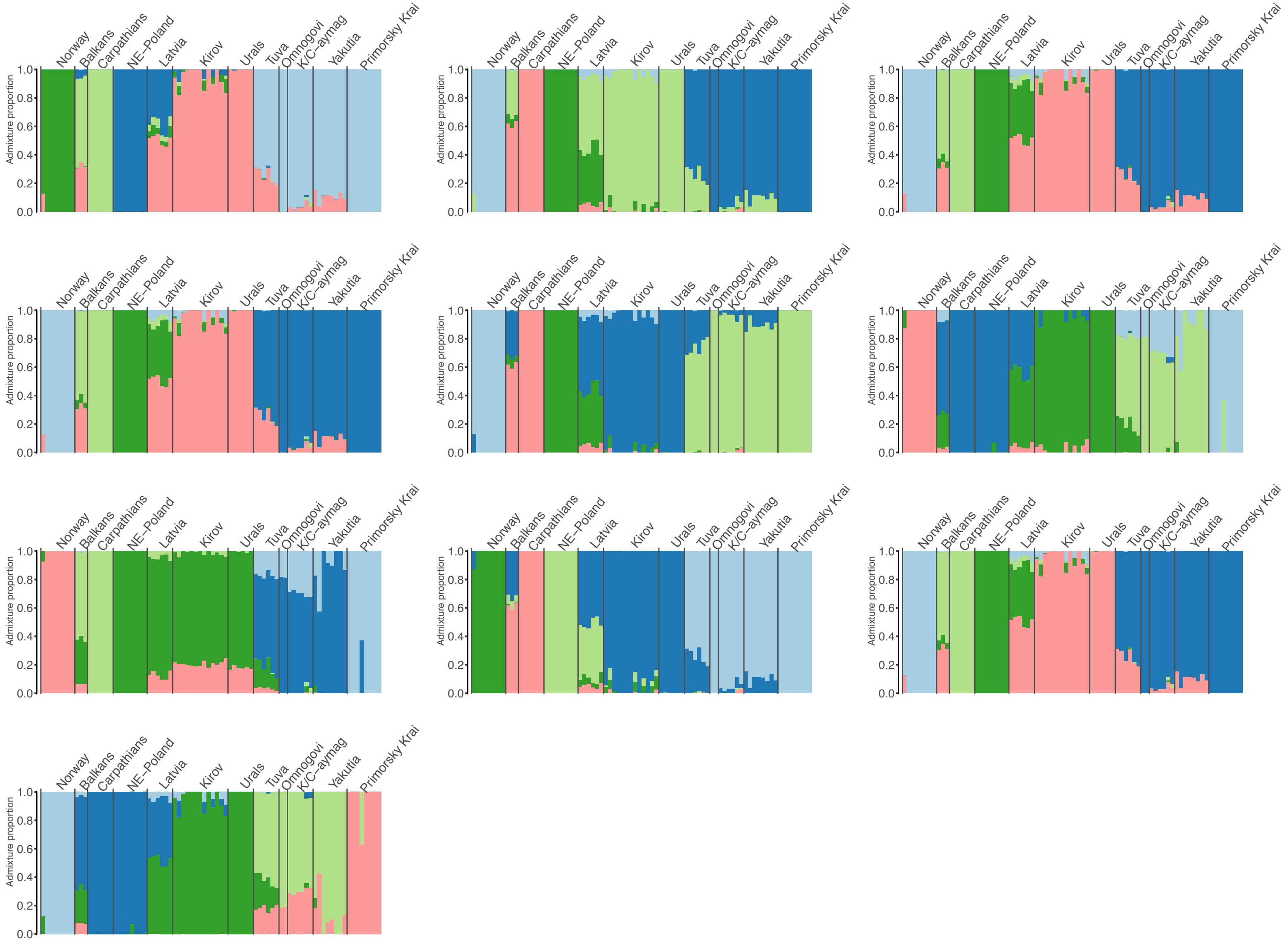

Fig. S11. Alternative clustering patterns obtained in 10 runs of NGSadmix for K=5. Mongolia population comprise K/C-aymag (i.e. Central and Khentii aymag) and Ömnögovi.

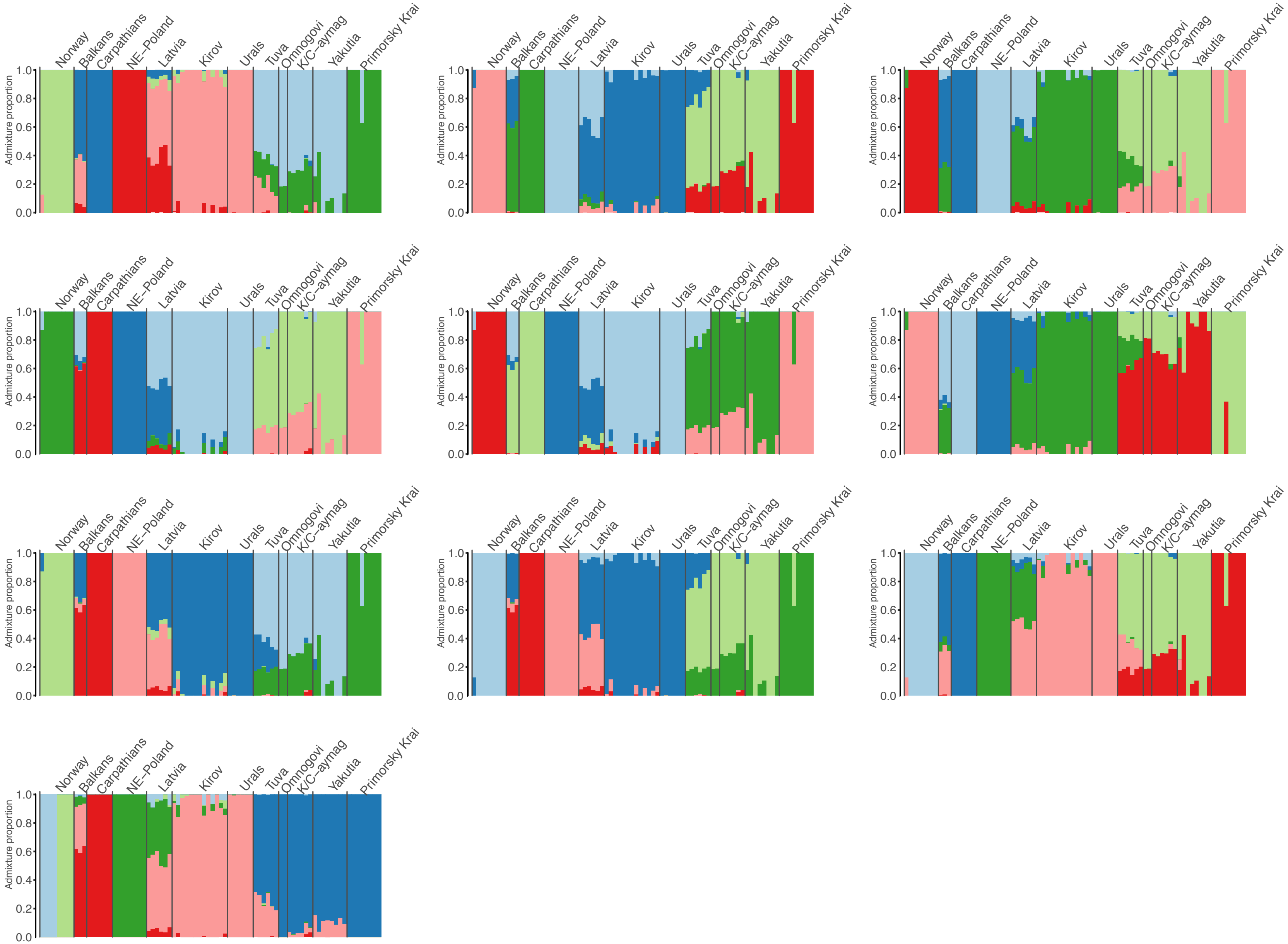

Fig. S12. Alternative clustering patterns obtained in 10 runs of NGSadmix for K=6. Mongolia population comprise K/C-aymag (i.e. Central and Khentii aymag) and Ömnögovi.

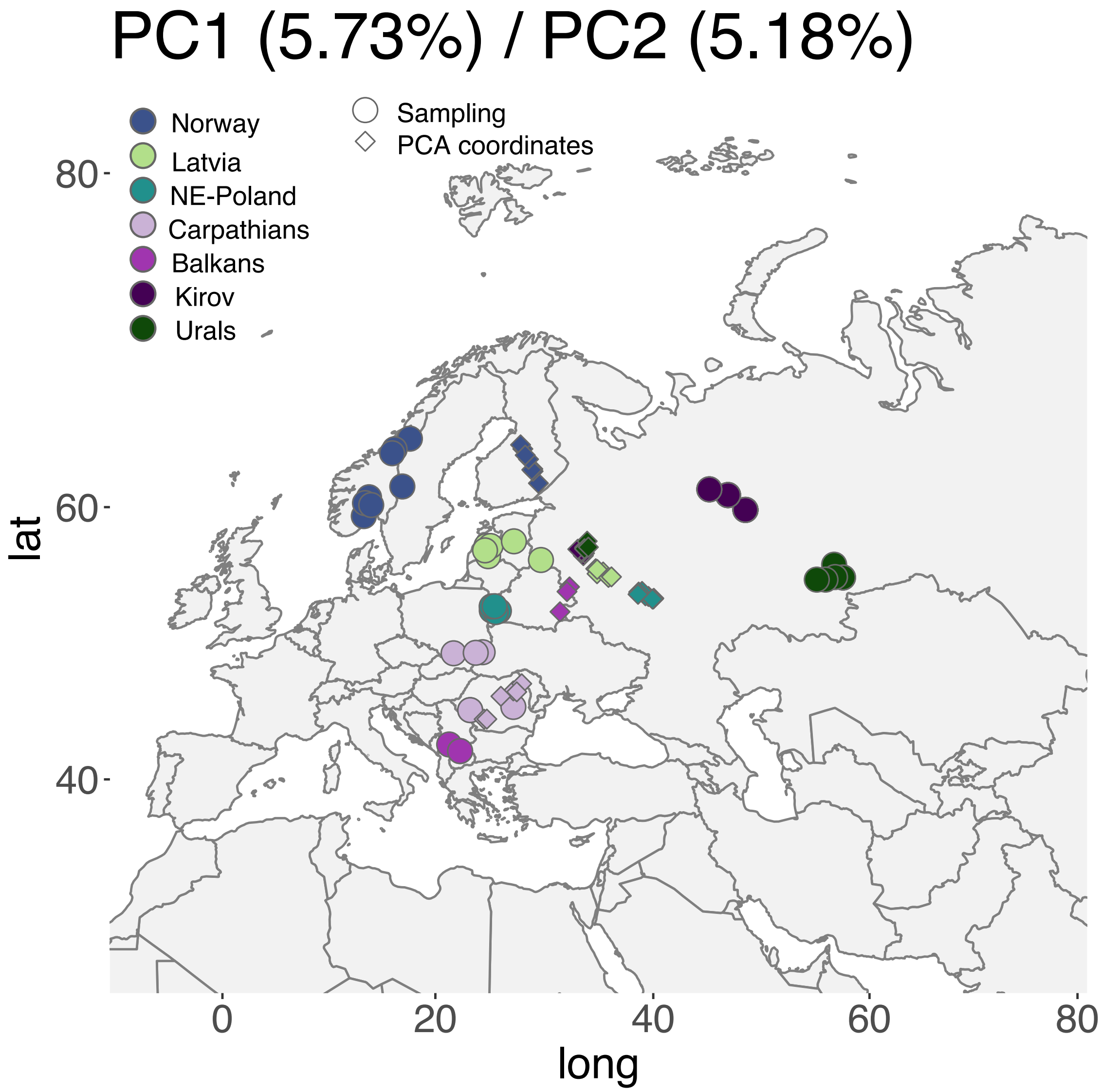

Fig. S13. Procrustes-transformed PCA coordinates to geographical coordinates in European populations, and sampling sites.

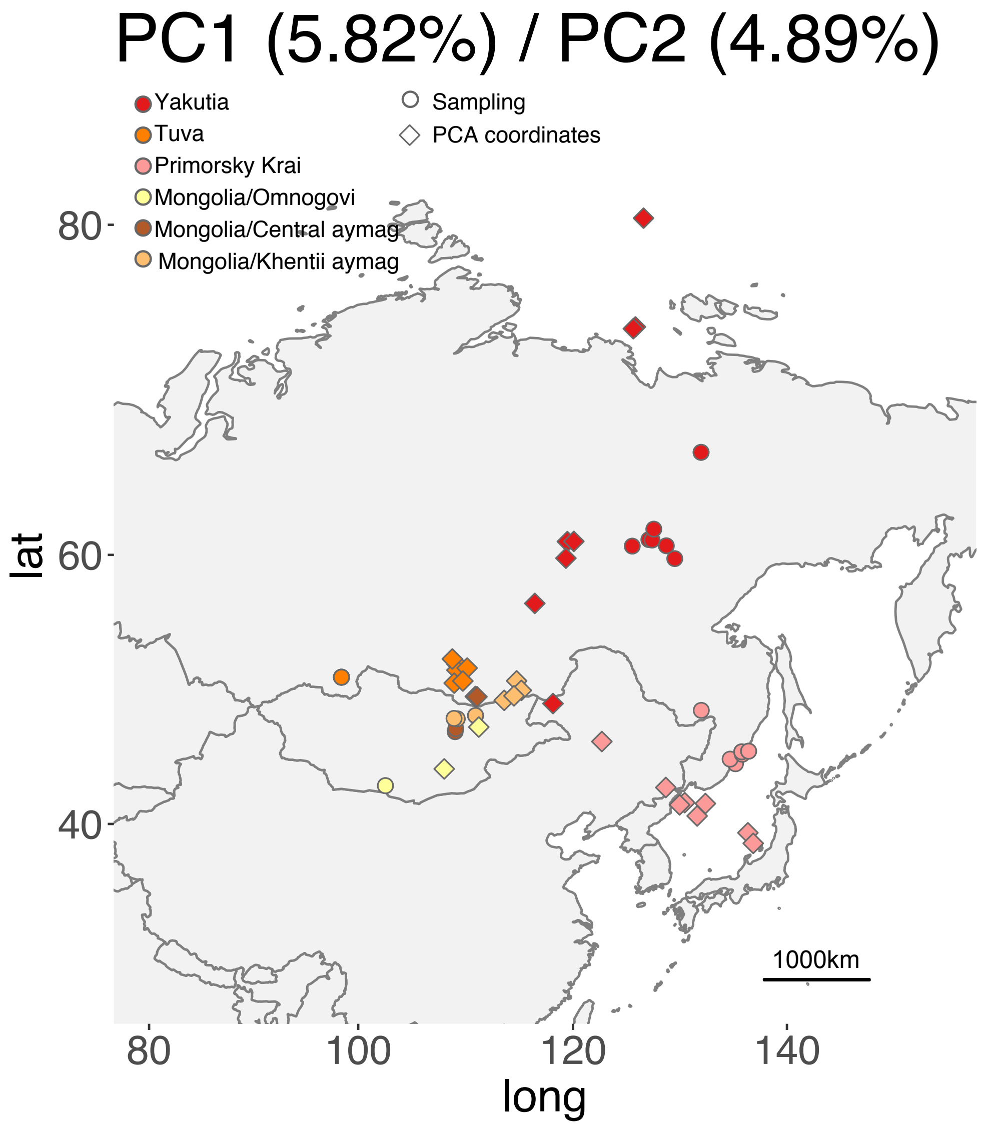

Fig. S14. Procrustes-transformed PCA coordinates to geographical coordinates in Asian populations, and sampling sites.

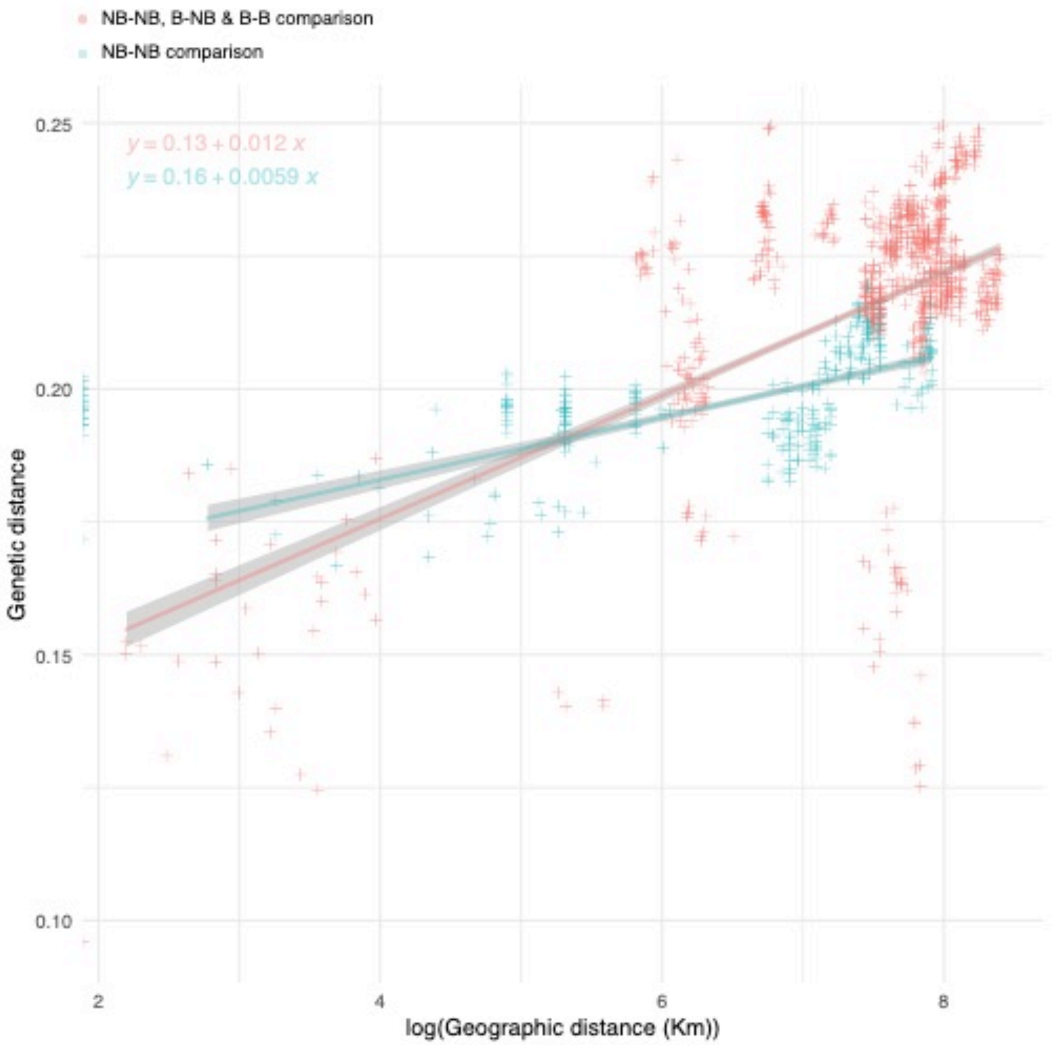

Fig. S15. Correlation of genetic vs. geographic distances between pairs of individuals from European populations, including and excluding individuals from European bottlenecked populations (Carpathians, Norway and NE-Poland). NB-NB: comparisons between two individuals from non-bottlenecked populations (NB); B-NB: comparisons between one individual from bottlenecked (B) and one individual from non-bottlenecked population (NB); B-B: comparison between two individuals from bottlenecked populations (B).

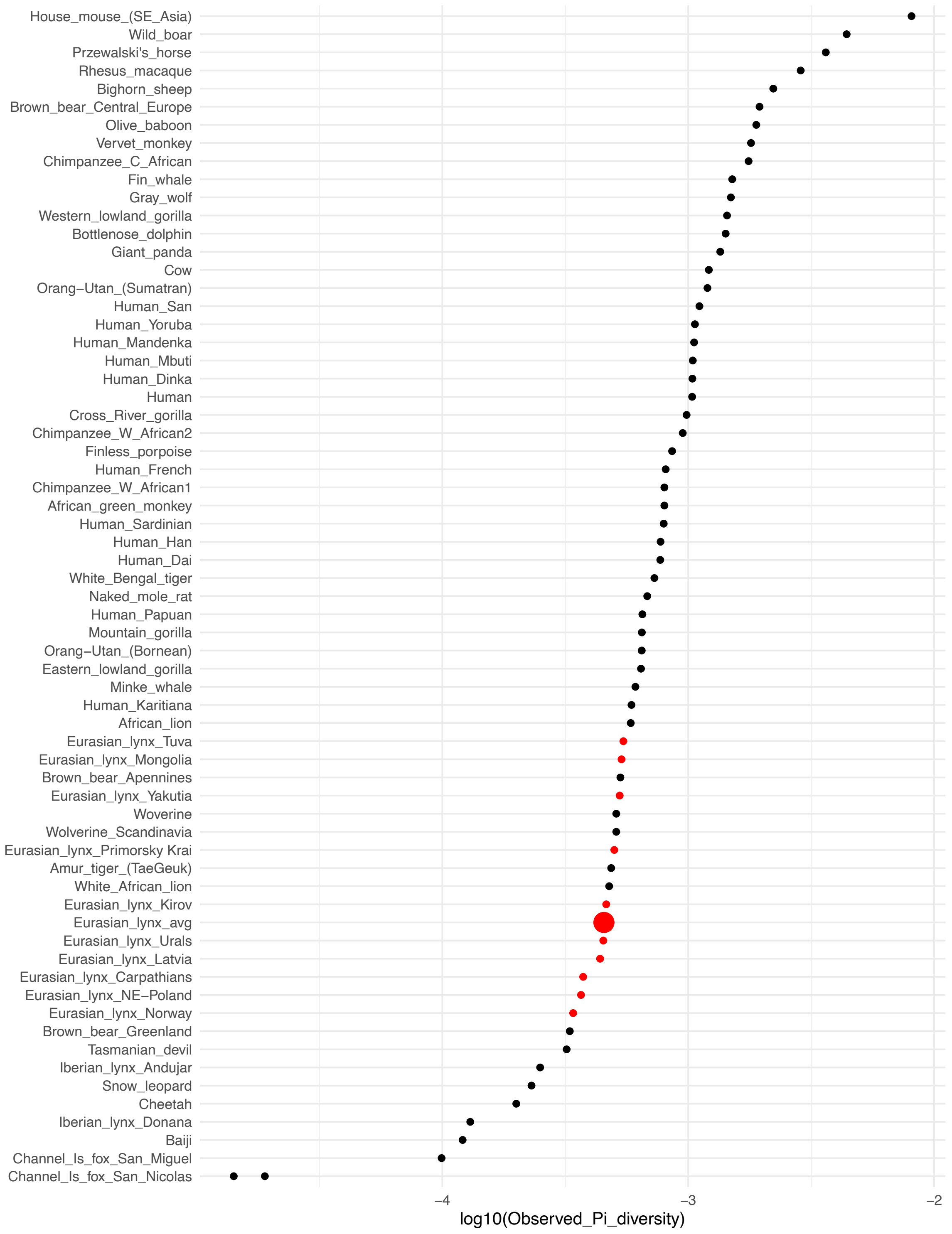

Fig. S16. Comparison of genomic nucleotide diversity among mammals. Modified from Robinson et al. (2016).

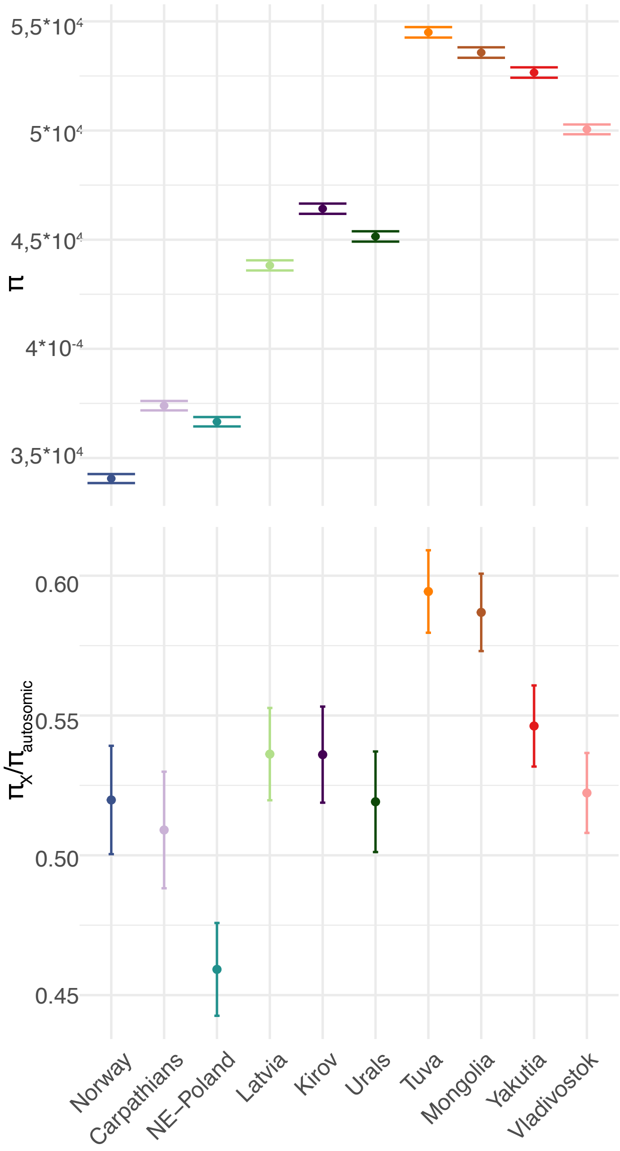

Fig. S17. Nucleotide diversity (π) values for autosomal sites and ratios of π diversity in Xchr vs. autosomes. Populations are sorted from west to east.

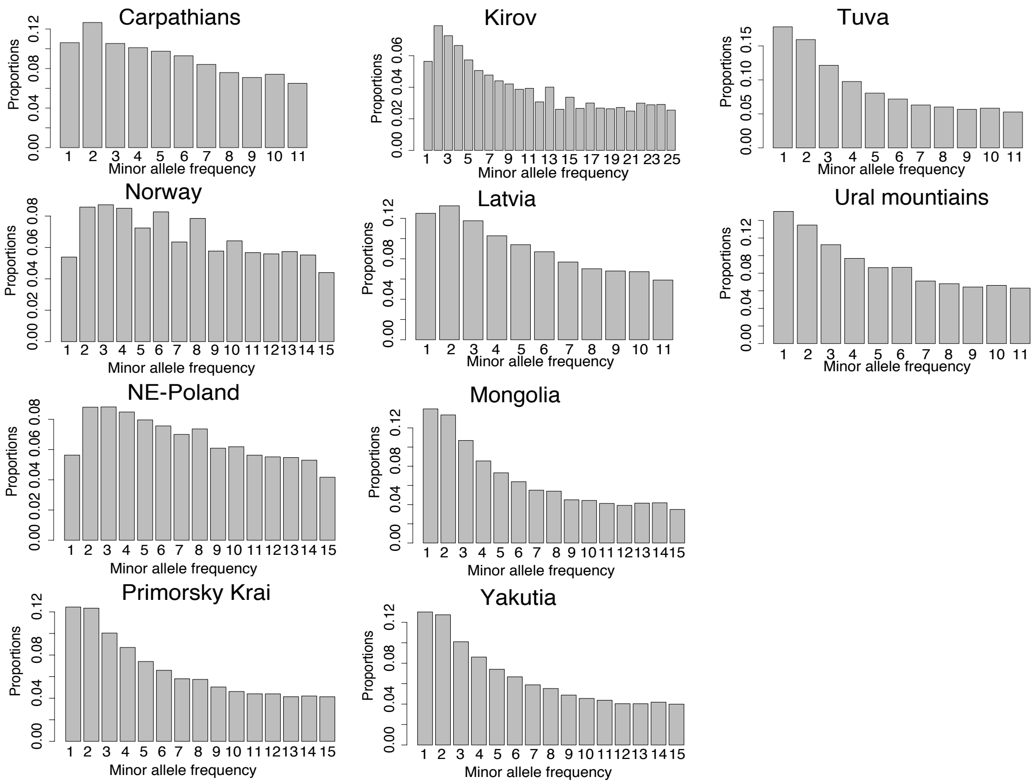

Fig. S18. Site frequency spectrum (SFS) of the different Eurasian lynx populations.

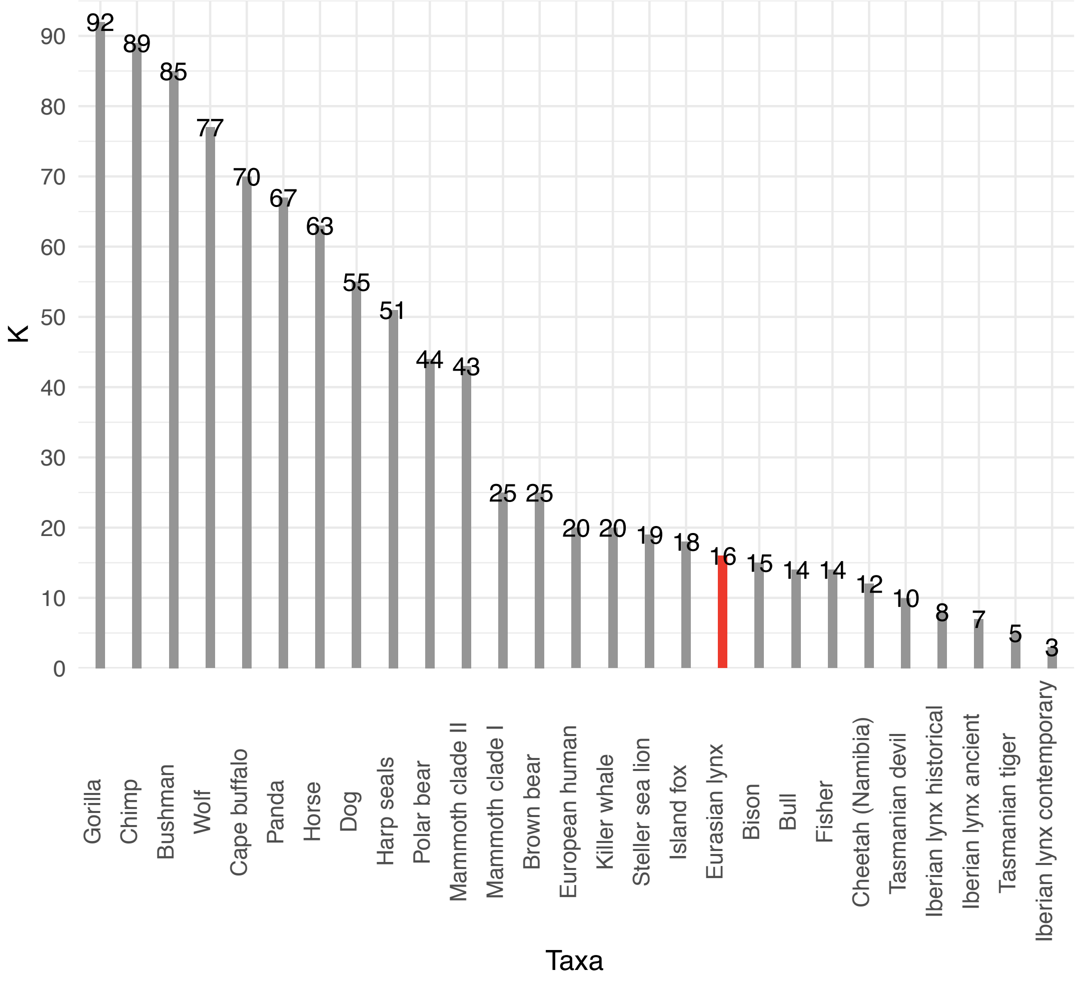

Fig. S19. Comparison of mitogenomic pairwise differences (k) among different species. Modified from (Casas-Marce et al., 2017).

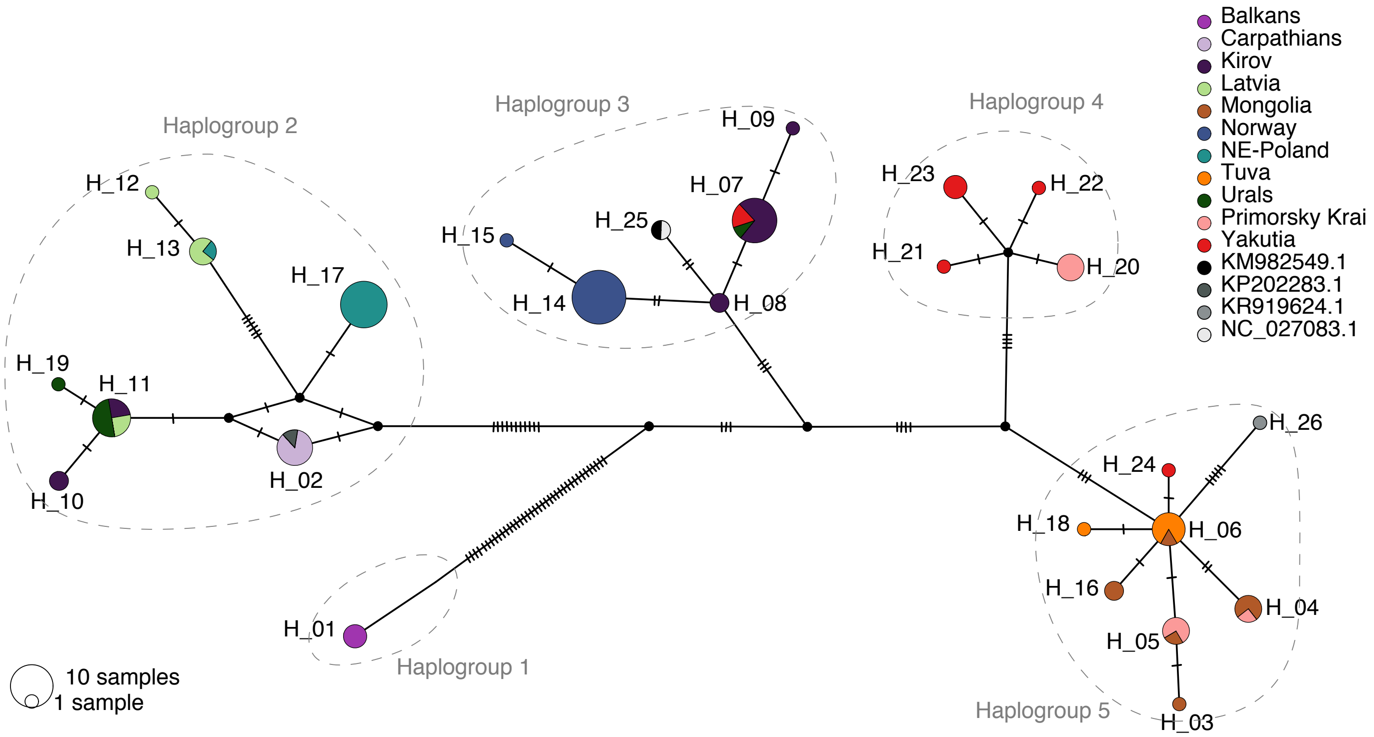

Fig. S20. Mitogenome haplotype network.

References

Abascal, F., Corvelo, A., Cruz, F., Villanueva-Cañas, J. L., Vlasova, A., Marcet-Houben, M., … Godoy, J. A. (2016). Extreme genomic erosion after recurrent demographic bottlenecks in the highly endangered Iberian lynx. *Genome Biology*, *17*(1), 251. doi: 10.1186/s13059-016-1090-1

Bouckaert, R., Heled, J., Kühnert, D., Vaughan, T., Wu, C.-H., Xie, D., … Drummond, A. J. (2014). BEAST 2: A Software Platform for Bayesian Evolutionary Analysis. *PLoS Computational Biology*, *10*(4), e1003537. doi: 10.1371/journal.pcbi.1003537

Brass, E. (1911). *Aus dem Reiche der Pelze* (I. & L. GmbH, ed.). Berlin, Germany.

Camacho, C., Coulouris, G., Avagyan, V., Ma, N., Papadopoulos, J., Bealer, K., & Madden, T. L. (2009). BLAST+: architecture and applications. *BMC Bioinformatics*, *10*, 421. doi: 10.1186/1471-2105-10-421

Casas-Marcé M, Marmesat E, Soriano L, *et al.* (2017) Spatio-temporal dynamics of genetic variation in the Iberian lynx along its path to extinction reconstructed with ancient DNA. *Molecular Biology and Evolution* **34**, 2893–2907.

DePristo, M., Banks, E., Poplin, R., Garimella, K., Maguire, J., Hartl, C., … Daly, M. (2011). A framework for variation discovery and genotyping using next-generation DNA sequencing data. *Nature Genetics*, *43*(5), 491–498. doi: citeulike-article-id:9134853\rdoi: 10.1038/ng.806

Garrison, E. P., & Marth, G. T. (2012). Haplotype-based variant detection from short-read sequencing. *ArXiv:1207.3907 [q-Bio.GN]*.

Hall, A. B., Qi, Y., Timoshevskiy, V., Sharakhova, M. V, Sharakhov, I. V, & Tu, Z. (2013). Six novel Y chromosome genes in Anopheles mosquitoes discovered by independently sequencing males and females. *BMC Genomics*, *14*, 273. doi: 10.1186/1471-2164-14-273

Kearse, M., Moir, R., Wilson, A., Stones-Havas, S., Cheung, M., Sturrock, S., … Drummond, A. (2012). Geneious Basic: an integrated and extendable desktop software platform for the organization and analysis of sequence data. *Bioinformatics (Oxford, England)*, *28*(12), 1647–1649. doi: 10.1093/bioinformatics/bts199

Kitchener, A. C., Breitenmoser-Würsten, C., Eizirik, E., Gentry, A., Werdelin, L., Wilting, A., … Tobe, S. (2018). A revised taxonomy of the Felidae. The final report of the Cat Classification Task Force of the IUCN/SSC Cat Specialist Group. *Cat News Special Issue 11*, 80.

Krojerová-Prokešová, J., Turbaková, B., Jelenčič, M., Bojda, M., Kutal, M., Skrbinšek, T., … Bryja, J. (2018). Genetic constraints of population expansion of the Carpathian lynx at the western edge of its native distribution range in Central Europe. *Heredity*. doi: 10.1038/s41437-018-0167-x

Kubala, J., Smolko, P., Zimmermann, F., Rigg, R., Tám, B., Iľko, T., … Breitenmoser, U. (2017). Robust monitoring of the Eurasian lynx Lynx lynx in the Slovak Carpathians reveals lower numbers than officially reported. *Oryx*, 1–9. doi: 10.1017/S003060531700076X

Lanfear, R., Frandsen, P. B., Wright, A. M., Senfeld, T., & Calcott, B. (2017). PartitionFinder 2: New Methods for Selecting Partitioned Models of Evolution for Molecular and Morphological Phylogenetic Analyses. *Molecular Biology and Evolution*, *34*(3), 772–773. doi: 10.1093/molbev/msw260

Li, H. (2013). Aligning sequence reads, clone sequences and assembly contigs with BWA-MEM. *ArXiv:1303.3997v1 [q-Bio.GN]*.

Li, H., Handsaker, B., Wysoker, A., Fennell, T., Ruan, J., Homer, N., … 1000 Genome Project Data Processing Subgroup. (2009). The Sequence Alignment/Map format and SAMtools. *Bioinformatics (Oxford, England)*, *25*(16), 2078–2079. doi: 10.1093/bioinformatics/btp352

Matyushkin, E. N., & Vaisfeld, M. A. (2003). *The lynx. Regional features of ecology, use and protection*. Moscow: Nauka.

McKenna, A., Hanna, M., Banks, E., Sivachenko, A., Cibulskis, K., Kernytsky, A., … DePristo, M. A. (2010). The genome analysis toolkit: A MapReduce framework for analyzing next-generation DNA sequencing data. *Genome Research*, *20*(9), 1297–1303. doi: 10.1101/gr.107524.110

Paijmans, J. L. A., Barnett, R., Gilbert, M. T. P., Zepeda-Mendoza, M. L., Reumer, J. W. F., de Vos, J., … Hofreiter, M. (2017). Evolutionary History of Saber-Toothed Cats Based on Ancient Mitogenomics. *Current Biology*, *27*(21), 3330-3336.e5. doi: 10.1016/j.cub.2017.09.033

Quinlan, A. R., & Hall, I. M. (2010). BEDTools: A flexible suite of utilities for comparing genomic features. *Bioinformatics*, *26*(6), 841–842. doi: 10.1093/bioinformatics/btq033

Rambaut, A., Drummond, A. J., Xie, D., Baele, G., & Suchard, M. A. (2018). Posterior Summarization in Bayesian Phylogenetics Using Tracer 1.7. *Systematic Biology*, *67*(5), 901–904. doi: 10.1093/sysbio/syy032

Robinson Jacqueline A, Ortega-Del Vecchyo D, Fan Z*, et al.* (2016) Genomic Flatlining in the Endangered Island Fox. *Current Biology* **26**, 1183-1189.

Sindičić, M., Gomerčić, T., Galov, A., Polanc, P., Huber, D., & Slavica, A. (2012). Repetitive sequences in Eurasian lynx (Lynx lynx L.) mitochondrial DNA control region. *Mitochondrial DNA*, *23*(3), 201–207. doi: 10.3109/19401736.2012.668894

Smeds, L., Warmuth, V., Bolivar, P., Uebbing, S., Burri, R., Suh, A., … Ellegren, H. (2015). Evolutionary analysis of the female-specific avian W chromosome. *Nature Communications*, *6*, 7330. doi: 10.1038/ncomms8330

Von Arx, M., Breitenmoser-Würsten, C., Zimmermann, F., & Breitenmoser, U. (2004). Status and conservation of the Eurasian lynx (Lynx lynx) in 2001. *KORA Bericht*, *19*.
